# Integrating Sustainable Hunting in Biodiversity Protection in Central Africa: Hot Spots, Weak Spots, and Strong Spots

**DOI:** 10.1371/journal.pone.0112367

**Published:** 2014-11-05

**Authors:** Julia E. Fa, Jesús Olivero, Miguel Ángel Farfán, Ana Luz Márquez, Juan Mario Vargas, Raimundo Real, Robert Nasi

**Affiliations:** 1 ICCS, Division of Biology, Imperial College London, Silwood Park Campus, Ascot, United Kingdom; 2 Universidad de Málaga, Grupo de Biogeografía, Diversidad y Conservación, Departamento de Biología Animal, Facultad de Ciencias, Campus de Teatinos s/n, Málaga, Spain; 3 Consultative Group on International Agricultural Research (CGIAR), CIFOR Headquarters, Jalan CIFOR, Situ Gede, Bogor, Indonesia; Institute of Agronomy, University of Lisbon, Portugal

## Abstract

Wild animals are a primary source of protein (bushmeat) for people living in or near tropical forests. Ideally, the effect of bushmeat harvests should be monitored closely by making regular estimates of offtake rate and size of stock available for exploitation. However, in practice, this is possible in very few situations because it requires both of these aspects to be readily measurable, and even in the best case, entails very considerable time and effort. As alternative, in this study, we use high-resolution, environmental favorability models for terrestrial mammals (*N* = 165) in Central Africa to map areas of high species richness (hot spots) and hunting susceptibility. Favorability models distinguish localities with environmental conditions that favor the species' existence from those with detrimental characteristics for its presence. We develop an index for assessing Potential Hunting Sustainability (*PHS*) of each species based on their ecological characteristics (population density, habitat breadth, rarity and vulnerability), weighted according to restrictive and permissive assumptions of how species' characteristics are combined. Species are classified into five main hunting sustainability classes using fuzzy logic. Using the accumulated favorability values of all species, and their *PHS* values, we finally identify weak spots, defined as high diversity regions of especial hunting vulnerability for wildlife, as well as strong spots, defined as high diversity areas of high hunting sustainability potential. Our study uses relatively simple models that employ easily obtainable data of a species' ecological characteristics to assess the impacts of hunting in tropical regions. It provides information for management by charting the geography of where species are more or less likely to be at risk of extinction from hunting.

## Introduction

Wildlife is a primary source of protein (bushmeat or wild meat) for many rural inhabitants in poor countries, particularly for people living in or near tropical forests [1]. However, unsustainable hunting of bushmeat can result in dramatic declines of local wild animal populations [2], [3]. The unsustainable harvest of mammals and birds can also have negative effects on forest structure and regeneration [4], ecosystem functioning [5], [6], and species diversity [7].

In West and Central Africa, many mammals (which include endemic and endangered species) are the main source of bushmeat protein in the region [4]. Due to the increase in human population, commercial trade of bushmeat has increased dramatically in the last three decades in these areas [2]. Such trade in wild animals for meat may have reached unsustainable levels, as the natural regeneration ability of wildlife populations may not be high enough to match the demand for bushmeat [2]. Hence, unsustainable extraction of wild meat in many tropical forests threatens the survival of a wide range of wildlife species as well as the food security of forest-dwellers [8]. However, areas that are more prone to species extinctions due to hunting are yet to be identified.

Empirical data on bushmeat harvest rates in large regions such as the Congo Basin are available for an increasing number of sites although these are still fragmentary [9]. So far these data alone cannot be used to advance strategies to mitigate the problem of wildlife exploitation and resolve food scarcity issues [2].

Bushmeat hunting sustainability has been defined and assessed most commonly via the use of indices [10], [11]. A number of sustainability indices have been published, and the production model (RR model) is the most commonly used [12]. The RR model employs literature values of a target species' carrying capacity and intrinsic population growth rate to calculate a maximum annual production, a fraction of which is then taken to be the species' maximum sustainable yield. Although the RR model has been applied to wildlife use studies at specific localities, it has also been used to assess production and extraction of bushmeat species at a landscape level [13]. A number of shortcomings have been noted in the application of production models to real-world situations [14].

Given that reliable monitoring of offtake (across all prey species at the necessary spatial and temporal scales) is notoriously hard, alternative methods to visualize hunting sustainability over large areas are urgently required. Species distribution modeling offers a mean for determining what environmental conditions are suitable for an animal or plant in geographic space [15]–[17], that can be coupled with classification of species according to some character of interest (e.g. their potential to withstand hunting pressure).

In this paper, we use favorability models to map the distribution of favorable areas for all hunted mammals in Central Africa. Topographic, hydrographic, climatic, land-cover, human and spatial variables are employed. Favorability modeling is a modality of species distribution modeling that reflects environmental favorability values rather than presence probability [18]. Favorability models have been successfully used for conservation purposes [19]–[22]. Then we combine the species' environmental favorability with their potential hunting sustainability to identify areas of high species diversity, as well as zones where future loss of wildlife is likely to be high, if hunting persists. We base sustainability on four species' ecological traits: population density, habitat breadth, rarity and vulnerability. This contribution is the first to present a hunting vulnerability map for bushmeat species in a large biodiversity-rich tropical region.

## Material and Methods

### Study Area

**Figure 1:**
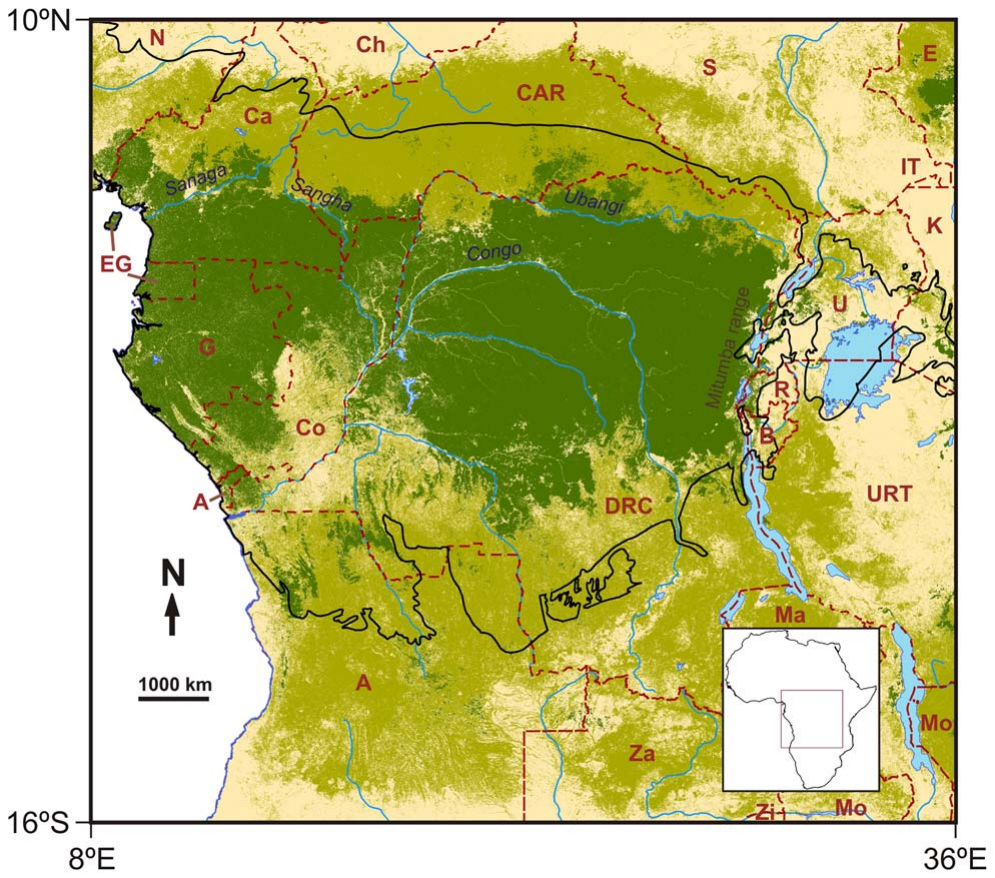
Map of the study region showing: rainforest (dark green), woody savannas (light green) extracted from [25]. The black line indicates the Rainforest Biotic Zone [27]. Countries: A Angola; B Burundi; Ca Cameroon; CAR Central African Republic; Ch Chad; Co Congo; DRC Democratic Republic of the Congo; E Ethiopia; EG Equatorial Guinea; G Gabon; IT Ilemi Triangle; K Kenya; Ma Malawi; Mo Mozambique; N Nigeria; R Rwanda; S Sudan; U Uganda; URT United Republic of Tanzania; Za Zambia; Zi Zimbabwe.

Our study area (10°N, 16°S, 8°E, 36°E) stretches from the coast of the Gulf of Guinea to the mountains of the Albertine Rift (Fig. 1) covering about seven degrees of latitude on either side of the Equator [23]. The central rainforest zone encompasses six main countries (the Democratic Republic of the Congo, the Republic of the Congo, Central African Republic, Cameroon, Gabon and Equatorial Guinea), as well as parts of another three (Angola, Burundi and Rwanda) (Fig. 1). The region contains the second largest and the least degraded area of contiguous moist tropical forest in the world, close to 2 million km^2^ [24]. The main vegetation types include evergreen/deciduous broadleaf forests and woody savannas, as well as areas of savanna and cropland-natural vegetation mosaic [25].

### Species Dataset

We first compiled a list of all terrestrial mammal species occurring within the geographical limits of our study region, using Kingdon *et al*. [26]. We also enumerated those species whose distributions overlapped with the Rainforest Biotic Zone, as defined by Kingdon *et al*. [26], following White [27], and inhabited habitats including rainforest. In order to select species for which there were records of being hunted for bushmeat, we used the list of species, recorded from the literature by Taylor et al. [28], with additional species included after consultation with another 4 experts working in the field. A total of 141 monotypic species and 24 other including 67 subspecies, belonging to 11 Orders, were finally used in our analyses (see Appendix S1 in File S1).

Distribution maps of all species in this list were downloaded as polygon shapefiles from the IUCN website [29] (maps compiled or modified in 2008). We considered only polygons of extant populations of the species that coincided with maps of those species in Kingdon *et al*. [26]. Polygons were then rasterized at a 0.01°×0.01° spatial resolution. The resulting raster maps were used to extract presence/absence values within a 1°×1° grid for the whole African continent.

### Species Distribution Modeling

“Extent of occurrence” range maps, such as those provided by IUCN, are only suitable for analysis at a maximum of 1°×1° spatial resolution [30]. This constraint can be overcome using distribution modeling and model downscaling [31]–[36]. We thus obtained maps describing distributions of environmentally favorable areas for species in 0.1°×0.1° resolution squares. Favorability models can show how the probability of a species' local presence differs from that expected by chance in the whole study area, and so can distinguish those localities with environmental conditions that favor the species' existence from those with detrimental characteristics for its presence [37]. In contrast to modeling techniques providing probability values, favorability models can distinguish between the effect of environmental conditions and the probability of presence derived from the species prevalence within the study area [37]. This enables direct comparison between models when several species are involved in the analytical design [37], and allows for model combinations through fuzzy logic [38], [39].

We built environmental favorability models for 141 species and 67 subspecies belonging to 24 other species. We attempted to develop independent models for every subspecies but environmental models were not found for most of them. This is because these subspecies have highly circumscribed distributions within which spatial autocorrelations predominated. Hence, we built a favorability model for each of the 165 species.

Models were executed for the entire African continent for the following reasons (see [40]): (1) some predictor factors considered in the models (climate, spatial historical constraints) required a large-scale modeling approach; (2) many species were broadly distributed throughout the continent, and so we had to include an environmentally significant geographical context for distinguishing between presences and absences; and (3) a large extent was required because we used a coarse spatial resolution. Model outputs, initially with a spatial resolution of 1°×1°, were later downscaled to 0.1°×0.1° resolution squares within our study area. For this, we employed the “direct downscaling approach” [33], classified by Bierkens et al. [32] as “downscaling based on mechanistic models through a deterministic [favorability] function”. Using the ArcGIS 10.0 raster calculator, the favorability model was thus projected to a 0.1°×0.1° resolution grid across the study area by applying the favorability equations to predictor variables at this resolution (see examples in [34]–[36]). A 10-fold shortening of the grain size (referring to pixel side length) does not severely affect predictions of species distributions [33], [41].

Models were built on the species' presence/absence in 1°×1° squares as the response variable, and were based on a list of 27 predictor variables describing topography, hydrography, climate, land cover/use and other indicators of anthropogenic pressure (Appendices S2 and S3 in File S1). Variables likely to have changed over time, such as farming, land cover and transport infrastructure, were taken for years within the decade before 2008, when species distribution maps were compiled or modified. A spatial descriptor was added to these variables to account for autocorrelation. This descriptor was defined for every species following the “trend surface approach” [42], and may account for the impact of dispersal barriers, geological history and biotic interactions. For this, a series of combinations of average longitude (Lo) and average latitude (La) for every square of the grid were entered in a stepwise logistic regression: Lo, La, Lo^2^, La^2^, Lo×La, Lo^3^, La^3^, Lo×La^2^, Lo^2^×La. The “trend surface variable” was then considered to be the resulting spatial *y*, i.e. the logit, or “*y*” lineal combination resulting from the logistic regression.

Type I errors, arising from the large number of variables used, were controlled using Benjamini & Hochberg's [43] procedure for controlling the False Discovery Rate (*FDR*). This control was performed before building each multivariate model, and we accepted only those variables that were significant under an *FDR* of *q*<0.05. In order to avoid multicollinearity, when the Pearson's correlation between two variables within a model was>0.8, only the variable most significantly predicting the species presence was retained.

Forward-backward stepwise logistic regression was run with the resulting set of variables [44], and probability outputs were finally transformed into favorability values [37]. The estimation of the relative weight of each variable in the model was tested using Wald's [45] test.

An alternative approach was used when the spatial-*y* had extremely high predictive value within the model. This was interpreted as a species distribution being constrained mostly by the spatial, possibly historical factor, and happened in two cases: 1) when the spatial-*y* was the only factor entered in the model; 2) when the Wald's parameter for the spatial-*y* was more than 10 times higher than the following variable in order of importance. In these cases, the niche theory [46], [47] advocates that the “realized ecological niche” of a species can be better explained by factors that imply spatial constraints on its distribution than by the ecological characteristics of the species itself. In these cases, a spatial model — based only on the spatial *y* — was intersected with an environmental model — in which the spatial *y* was not considered — using the fuzzy intersection. This intersection describes simultaneous spatial and environmental favorability for the presence of the species [39]. The fuzzy intersection was calculated as the minimum favorability value in any of the two models [48].

Highly favorable sites where a species can be present are possible outside their current distribution ranges [47]. In our analysis, we derived favorability values for the species only where it is known to occur, because we were interested on how sustainable present populations are likely to be. Thus, species can persist within their current distribution area if populations of that species are sustainable. The distribution areas for subspecies were considered separately, hence a total of 208 species maps of 141 monotypic species and 67 subspecies were obtained.

### Describing a Species' Potential Hunting Sustainability

In this study, we used the fuzzy logic approach to avoid subjective thresholds when describing a species' “Potential Hunting Sustainability” (*PHS*), that is, the species' potential resilience to hunting according to ecological traits that are linked with extinction proneness [49]. The logic behind fuzzy sets states that the membership of any element to a set is neither completely true nor false, whereas a membership function, assigning to each element a real number in the interval [0, 1], describes the degree to which it meets the definition of the set [48]. Thus, the fuzzy approach allowed to consider all species as members of the set of species whose hunting is sustainable, each one having, however, a different degree of membership.

The first step for estimating *PHS* was to calculate, for each taxon, a “Sustainability Index” (*SI*) based on its population density weighted by a combination of other ecological traits - habitat breadth, rarity and vulnerability (see below). *SI* was calculated by considering two different fuzzy-logic operations:

Fuzzy union
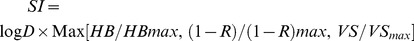
(1)

Fuzzy intersection
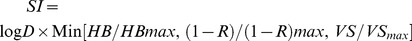
(2)where *D* is population density, *HB* is habitat breadth, *R* rarity and *VS* vulnerability status of a given species. *D* was log-transformed for linearizing its highly pronounced exponential behavior. *SI* increases with all these traits, hence 1-rarity, and not rarity, is used. The fuzzy union allowed a “permissive” approach for incorporating the relevance of the three later factors in *SI*, i.e. a high value in a single factor enabled a high weighing of log*D*. Instead, the fuzzy intersection related to a “restrictive” weighting in which high values are required in the three factors for a high weighting of log*D*.

We derived population densities for all taxa in our list from various sources: data for 53 (32%) species directly as in PanTHERIA [50]; 15 (9%) as in Fa & Purvis [51]; and 97 (59%) from the expected values derived from a linear regression of log(population density) on the basis of log(body mass), performed by us using the worldwide data in PanTHERIA (*N* = 949 species, *R*^2^ = 0.5743, *P*<0.05). Habitat breadth was defined as the number of main habitats occupied by a taxon. Ten habitats were considered: forest, fragmented forest, forest-savanna/pasture mosaic, woody savanna, savanna/pasture, scrubland, bareland, moorland, mangrove and farmland; we scored each taxon with one point per occupied habitat according to Kingdon et al. [26]. Rarity reports on the size range of each species and was measured as 1 - the proportion of the total surface area of the African continent occupied by the taxon from distribution data from IUCN [29]. ArcGIS 10.0 Raster Calculator was employed. Vulnerability of a taxon was the conservation status category according to the IUCN Red List. We distributed points in this way: 0 =  Critically Endangered, 1 =  Endangered, 2 =  Vulnerable, 3 =  Near Threatened, 4 =  Least Concern. We used the latest version of the IUCN Red List [29].

We then estimated the Potential Hunting Sustainability (*PHS*) score of each taxon:
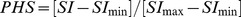
(3)

*PHS* is, in practice, a rescaling of *SI* in the interval [0, 1]. The essential tenet of this index is based on the observation that body size is inversely correlated with population density, making large-bodied animals less abundant and more vulnerable to human activities like hunting [49]. The extinction proneness of large-bodied animals is further enhanced because of other correlated traits, such as their requirement of large area, greater food intake, high habitat specificity, and lower reproductive rate. Species in our data set include taxa where hunting is more sustainable, mostly the smaller species, and species that are more extinction prone from hunting, the larger-bodied species. Thus, *PHS* ranged from 0 when sustainability equaled the minimum value observed in any species of our data set [*SI* = *SI*_min_] to 1 when sustainability equaled the maximum value observed [*SI* = *SI*_max_].

### Fuzzy Sets for Mapping Hunting Sustainability

Maps representing the favorability for every species/subspecies within their distributions were integrated by employing an index hereinafter referred to as the “Accumulated Favorability” (*AF_j_*), which constitutes a surrogate of biodiversity [38]. High values of this index represent fuzzy favorability hot spots, and have been considered in the assessment of site networks for the protection of biodiversity [38]. The accumulated favorability is the result of adding up the favorability (*F_i_*) value for all *i* taxa in each *j* cell in the study area:
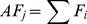
(4)

We obtained a measure of “Sustainable Accumulated Favorability” (*SAF_j_*) by weighting *F*, in equation 4, according to the *PHS* as defined in equation 3:
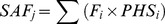
(5)

We also calculated the “Unsustainable Accumulated Favorability” (*UAF_j_*) by weighting *F_i_* according to 1-*PHS_i_*:
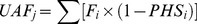
(6)

Both *SAF_j_* and *UAF_j_* are complementary indices the sum of which equals *AF_j_*. Theoretically, these three indices could range from 0 to the number of species included in the analysis. Just like *AF_j_* represents the total diversity of hunted mammals, *SAF_j_* quantifies the diversity of species of high hunting sustainability potential; instead, *UAF_j_* quantifies the diversity of highly vulnerable species to hunting. This complementarity is, thus, consistent with the geographical overlap of high *SAF_j_* and high *UAF_j_* areas, because vulnerable and resilient species to hunting can coexist. The fuzzy logic approach allowed us, however, to avoid subjectively classifying species as sustainable or unsustainable. This way of weighting the constituents of a diversity index (in our case, *F_i_*) with a factor representing degrees of fuzzy membership (in our case, in the set of species whose hunting is sustainable), has been a successful procedure as demonstrated in Olivero et al. [52], [53].

Geographical hot spots (areas of high species richness), strong spots (high diversity areas of high hunting sustainability potential) and weak spots (high diversity regions of especial hunting vulnerability for wildlife) were defined by selecting grid cells with the highest 5% of *AF_j_*, *SAF_j_* and *UAF_j_* values, respectively. This arbitrary cutoff was selected to match the proportion of our study area that is currently protected within rainforest reserves according to the World Database on Protected Areas [54]. This threshold was also used in Cardillo et al. [55] and Estrada et al. [38].

### Defining Sustainability Categories

**Figure 2:**
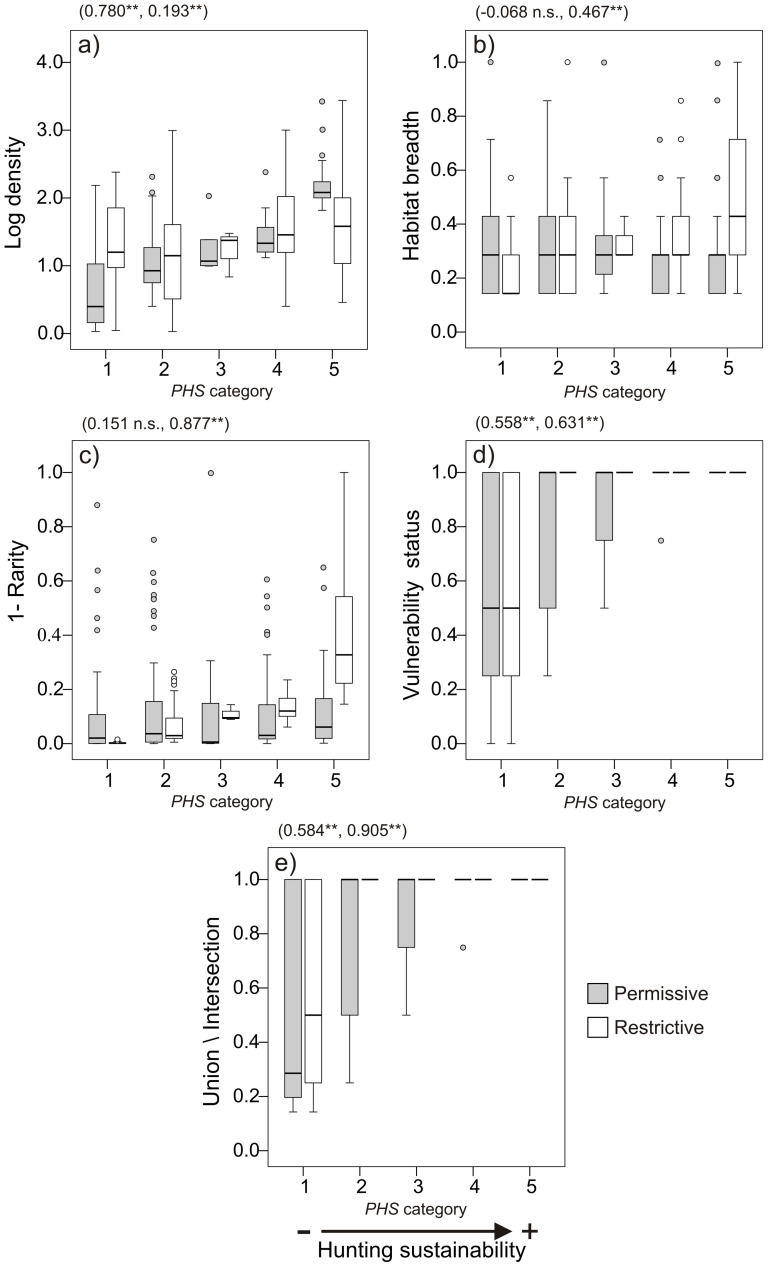
Boxplots linking potential hunting sustainability *(PHS)* categories and species' ecological traits used to calculate *PHS*. Traits considered are: a) log-transformed population density; b) habitat breadth; c) 1-rarity; d) vulnerability status; e) union and intersection of habitat breadth, 1-rarity and vulnerability status (combinations driving the permissive and the restrictive approaches, respectively). *PHS* increases with all traits. Spearman correlations, in brackets, are shown between *PHS* and each sustainability factor for the permissive and restrictive weighting (** = *P*<0.01).

**Figure 3:**
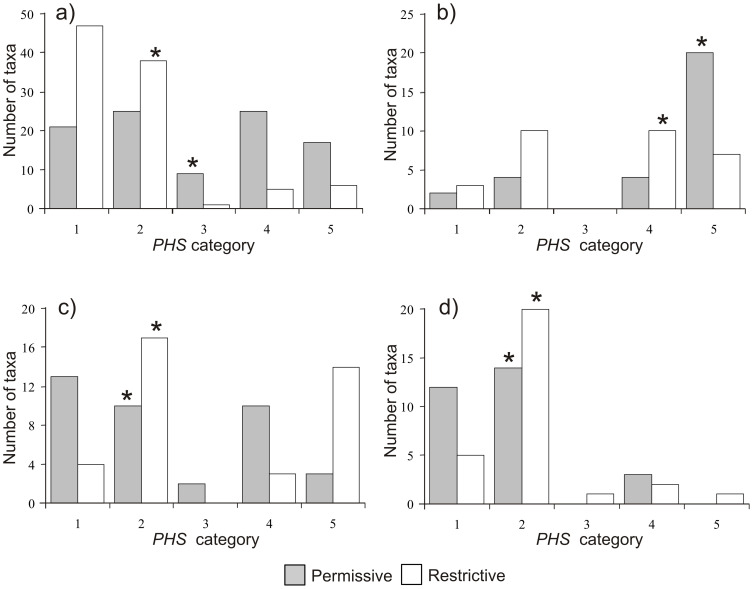
Number of taxa associated with the five potential hunting sustainability (*PHS*) classes. **a**) Primates; **b**) Rodentia; **c**) Carnivora; **d**) Cetartiodactyla. Asterisks indicate medians. Sustainability increases from class 1 to 5.

Once species and subspecies were ordered according to *PHS* (equation 3), we divided the list into five taxon clusters representing categories of sustainability (1 =  minimum sustainability and 5 =  maximum sustainability. Our purpose here was to facilitate the interpretation of our results (see Figs. 2 and 3), without using these categories as fixed classifiers of sustainable hunting. Cutoffs for the central category were based on the standard deviation of the mean *PHS* (mean *PHS*± standard error). We calculated the cutoff for the highest sustainability category by accumulating *PHS*, from the highest to the lowest value, until the maximum local *SAF_j_* (equation 5) observed within our study areas was reached. This threshold allowed the grouping of species whose accumulation would equal the maximum observed *SAF_j_*, should completely favorable areas for all of them overlap geographically. For the lowest sustainability category, *PHS* was accumulated from the lowest to the highest value until the maximum local *UAF_j_* (equation 6) was reached. Two sets of sustainability categories were then developed, depending whether the fuzzy union (equation 1) or the fuzzy intersection (equation 2) was applied to calculate *SI*.

## Results

### Potential Hunting Sustainability (*PHS*)

For all taxa, we constructed separate listings of the *PHS* values derived for the permissive or restrictive weighting (Appendix S1 in File S1). We found that for the permissive weighting, *PHS* was significantly positively correlated with species population density (*R*^2^ = 0.780; *P*<0.01) and then with their vulnerability status (*R*^2^ = 0.558; *P*<0.01) (Fig. 2). Thus, species likely to be unsustainable in the permissive weighting were those with low population densities, but also taxa that were threatened even having a relatively higher abundance. In contrast, *PHS* values for the restrictive weighting were significantly associated with species' rarity (*R*^2^ = 0.877; *P*<0.01), followed by their vulnerability status (*R*^2^ = 0.631; *P*<0.01) and habitat breadth (*R*^2^ = 0.467; *P*<0.01). Unsustainability here was related to small home ranges, threat status, and by a more limited habitat breadth (Fig. 2).

The distribution of all Central African mammals (*N* = 208) differed significantly by *PHS* category according to whether we applied permissive or restrictive weightings to calculate the *PHS* (Fig. 3, Appendix S4 in File S1). A total of 51.4% of taxa belonged to the low *PHS* categories (1 and 2) according to the permissive weighting, but this proportion was higher (72.6%) for the restrictive weighting. In contrast, 42.8% and 26.0% of all taxa were included in the high sustainability categories (4 and 5) according to the two criteria, respectively.

For the four most represented mammalian orders, clear differences between *PHS* classes appeared in the frequency distribution of taxa (Fig. 3). For primates, *PHS* was skewed towards the less sustainable classes (1 and 2) for the strict weighting criterion, but was more evenly distributed in the permissive approach. By comparison, most Rodentia were found within the two most sustainable categories (4 and 5) regardless of the weighting used. No significant groupings were found for Carnivora although there was a slight tendency towards least sustainable *PHS* categories. Finally, most Cetartiodactyla were grouped around *PHS* categories 1 and 2, regardless of the weighting used.

### Weak Spots, Strong Spots and Hot Spots

Favorability models were obtained for the 165 mammal species included in our analyses. Only two variables, “Forest” and “Intact Forest”, showed Pearson's correlations>0.8; thus, we avoided these to enter together in the same model. The distributions of 19% of the species were explained mostly by the spatial factor, possibly denoting historical constraints; in these cases, the intersection of a purely spatial and a purely environmental model provided complete environmental favorability models. Finally, 208 favorability maps with a 0.1°×0.1° spatial resolution were obtained: 141 for monotypic species and 67 for subspecies. All maps were integrated with each other according to the formulas defining accumulated favorability values (equations 4–6).

**Figure 4:**
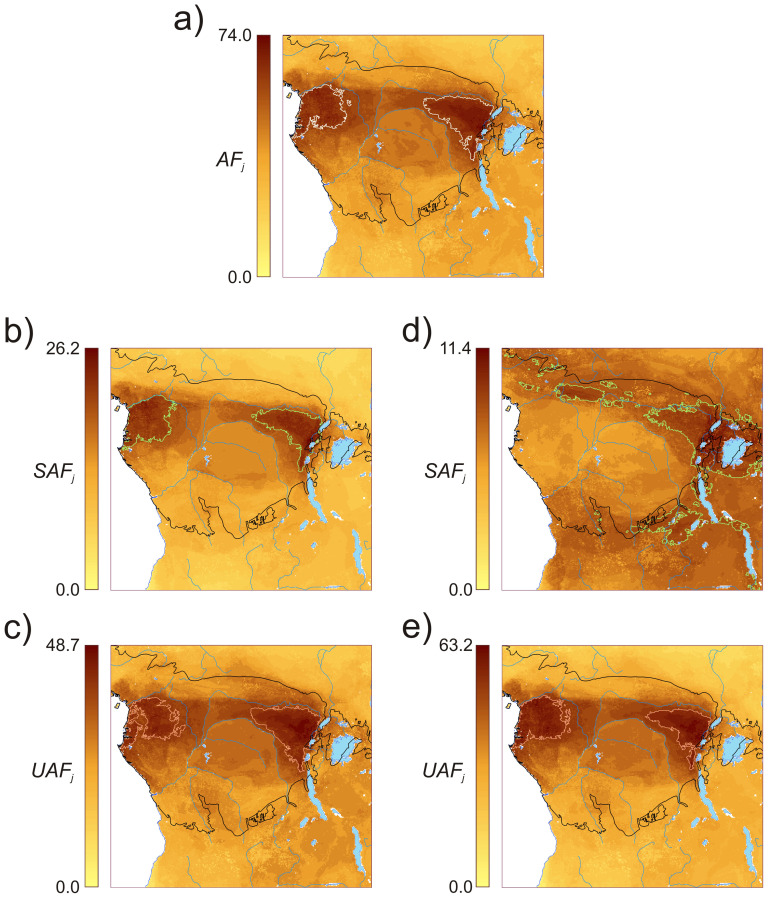
Distribution of hot spots, strong spots and weak spots in the study region. **a) Hot spots** derived from the *Accumulated Favorability* (*AF_j_*) of all mammals (208 taxa) in the analysis (*AF_j_* range: 60.1–74.0). **b) Strong spots (permissive)**, *Sustainable Accumulated Favorability* (*SAF_j_*) according to the permissive weighting of the potential hunting sustainability (*PHS*) (*SAF_j_* range: 20.1–26.2). **c) Weak spots (permissive)**, *Unsustainable Accumulated Favorability* (*UAF_j_*) according to the permissive weighting (*UAF_j_* range: 40.0–48.7). **d) Strong spots (restrictive)**, *Sustainable Accumulated Favorability* (*SAF_j_*) according to restrictive weighting (*SAF_j_* range: 8.6–11.4). **e) Weak spots (restrictive)** *Unsustainable Accumulated favorability* (*UAF_j_*) according to the restrictive weighting (*UAF_j_* range: 53.5–63.2). White lines outline hot spots, pale green lines outline strong spots, and pink lines outline weak spots. The black line delimits the Rainforest Biotic Zone [27].

Maps representing the Accumulated Favorability (*AF_j_*) values for all taxa show that the highest values were found within the main rainforest block between the Albertine Rift and the Atlantic Ocean (Fig. 4a), but both north of the Congo River. Two biodiversity hot spots are clear, one in the northwest in the study area stretching from the Atlantic coast north to the Sanaga River and east towards the Sangha and Congo Rivers. The second hot spot nestles in the eastern most part of the study area, West of the Mitumba Mountain range in the Western Rift Valley (Democratic Republic of Congo), and to the south of the upper course of the Ubangi River.

**Figure 5:**
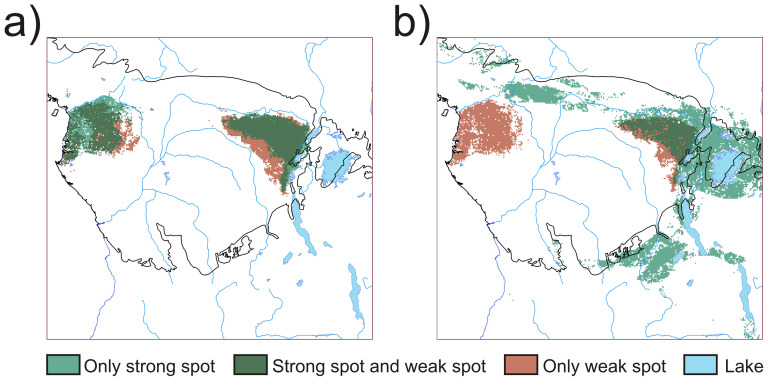
Maps showing the spatial overlap between weak spots and strong spots. **a**) permissive weighting; **b**) restrictive weighting. Weak spots are areas of highest unsustainable accumulated favorability, and strong spots are areas of highest sustainable accumulated favorability.

Sustainable and unsustainable accumulated favorability for the permissive criterion were located within the rainforest region where hot spots, strong and weak spots largely coincide (90.2% of weak spots and 88.8% of strong spots are also hot spots, Figs. 4b and 4c). The coincidence is almost perfect in the case of the weak spots, though the eastern strong spot boundaries are slightly contracted northward along the southern parts. Distribution of weak, strong and hot spots for the restrictive approach show that weak spots, not strong spots, overlap with hot spots (97.1% of weak spots but only 56.3% of strong spots are also hot spots, Fig. 4c). Strong spots and hot spots coincide in the eastern part of the study area; strong spots spread eastward along rainforest areas east of the Mitumba Mountains, but also occupy crop mosaic, woody savanna and grassland habitats around Lake Victoria, as well as woody savannas to the north and south (Fig. 4d). Maps showing the overlap between strong spots and weak spots defined by the permissive and restrictive approaches are shown in Fig. 5a and 5b, respectively.

For both permissive and restrictive approaches, our results showed that most taxa (over 97%) within the most sustainable classes (i.e. 4 and 5) were those of Least Concern [*VS*/*VS*_max_ = 1, Appendix S1 in File S1], with the remaining 3% being Vulnerable [*VS*/*VS*_max_ = 0.75]. In contrast, around 65% of taxa in the least sustainable categories (classes 1 and 2), were Near Threatened, Endangered or Critically Endangered. Moreover, almost all (92%) of the rainforest taxa [*HB*/*HB*_max_ = 0.14, Appendix S1 in File S1] were classified within the least sustainable classes in the restrictive approach, whereas only 54% of these were included in these classes in the permissive approach.

Weak spots contained a high concentration of high conservation value taxa like western lowland gorilla *Gorilla gorilla gorilla*, eastern lowland gorilla *G. beringei graueri*, the two subspecies of chimpanzee *Pan troglodytes*, Adolf Friedrichs's Angola colobus *Colobus angolensis ruwenzorii*, golden-bellied crowned monkey *Cercopithecus pogonias pogonias*, owl-faced monkey *C. hamlyni*, Western putty-nosed monkey *C. nictitans martini*, L'hoest's monkey *Allochrocebus lhoesti*, okapi *Okapia johnstoni,* forest elephant *Loxodonta cyclotis* and savanna elephant *L. africana*. However, more than half of the Near Threatened, Endangered and Critically Endangered taxa lay outside the limits of our weak spots; over 80% of these have highly restricted distributions [(1-*R*)/(1-*R*)_max_<0.01, see Appendix S1 in File S1]. This group included Critically Endangered mammals like the mountain gorilla *Gorilla beringei beringei*, Cross River gorilla *G. gorilla diehli*, Schouteden's blue monkey *Cercopithecus mitis schoutedeni*, Dryad monkey *C. dryas*, Bouvier's red colobus *Procolobus pennantii bouvieri* and Preuss's red colobus *P. preussi*.

## Discussion

### Use of spatial modeling in hunting sustainability

Some studies have used direct estimates of carrying capacity of catchment areas and actual or predicted population densities to establish hunting sustainability [56]. Such high quality empirical data can inform better models, but these are currently not available for large-scale projections. These data limitations typically mean that only simpler models can be generated presently. Although better data on local species composition and densities of individual species are becoming available [57], [58], the urgency of the problem of overhunting in the tropics means that heuristic tools are useful to offer an immediate solution, even if not optimal.

Our study is the first to use spatial modeling tools for assessing geographical distributions of hunted mammals at a large scale. We employed favorability models to assess ecological responses of species to environmental conditions. These models differ from other modeling approaches since they do not reflect presence probability, but rather environmental favorability values, which are of greatest interest to distribution modelers [18], [37]. Unlike probabilities, favorability describes local deviations from the overall probability of presence; this provides a model output that is independent from the species' prevalence, which allows models of different species to be compared and combined. The *F_i_* value may be considered as the degree of membership of the fuzzy set of areas favorable for species *i*, so that it may be used to apply the concepts, operations and rules of fuzzy logic to environmental modeling: for example, 1−*F_i_* corresponds to the degree of membership of the complementary fuzzy set of sites whose environmental conditions are unfavorable to the species. These values also allow for directly comparing the degree of favorability, for instance, of sustainable and unsustainable taxa, which is more difficult to achieve using the original logistic functions, as the different proportions of presences for the two species bias their random expectations in opposite directions. A region may be equally favorable for both species, even if one of them is much less frequent due to its biology or behavior. Favorability models are useful to elucidate biogeographical trends, as well as for practical purposes such as the selection of the most suitable locations for species reintroductions.

In this paper, we developed a new approach in which we combined models defining local environmental favorability for hunted species with their potential for a sustainable hunting. Our index essentially draws from a considerable body of research relating to how intrinsic characteristics of mammals [49] can be used to derive a measure of their vulnerability to hunting. In particular, we focus on the negative relationship between body mass and ecological characteristics (population density, reproductive rates) based on the observations that large-bodied mammals are most at risk from hunting [59], and are often the preferred by hunters [60]. Thus, we employed the actual or derived population density estimates for each species as the basis for our hunting sustainability index.

We used different weightings for quantifying *PHS* as a guide to provide policy makers with the choice of two different set of criteria at varying levels of “zeal” i.e. a more lenient “one criterion is sufficient” permissive approach vs. a sterner restrictive one “all criteria must be enforced” (see equations 1 and 2). The permissive approach was clearly influenced principally by species population density and vulnerability status, whilst the restrictive one was mostly linked to rarity. These effects are not *ad hoc* but are explicable by the nature of the weightings we employed. Moreover, the restrictive approach classified more species within the lowest *PHS* categories (i.e. taxa at greater risk of overhunting) compared with the permissive approach. This means that the difference between both criteria is also qualitative, since species appear ordered in distinct ways in both lists (Appendix S1 in File S1). This weighting-based differential ordering of species also resulted in the identification of distinct geographical locations for strong spots. Thus, in the permissive weighting, weak and strong spots widely overlapped within the rainforest region (Fig. 5a), whereas for the restrictive weighting sustainable diversity moved towards extensive ecotonal regions between the rainforest and more open lands in the East (Fig. 5b). This is a result of the restrictive weighting considering almost all forest-bound species as unsustainable (93% of the 73 rainforest taxa in classes 1 and 2) and thus delimiting strong spots outside the rainforest block. In contrast, 42% of the forest-bound taxa (i.e. 33 species and subspecies) were classified amongst the most sustainable classes by the permissive approach, contributing to strong spots within the rainforest area.

### Use of IUCN species distribution maps

We used the species distribution range maps published by the IUCN as the basis of our analyses. Favorability models based on these may have some limitations, which we have tried to overcome by: (1) training the models by employing a spatial resolution at which ‘extent of occurrence’ range maps are still informative [30]; (2) downscaling models to a spatial resolution for which high quality environmental data are widely available (see Appendix S2 in File S1); and (3) applying only a 10-fold shortening of the grain size, which should not severely affect predictions of species distributions [33], [41]. Moreover, we account for the impact of dispersal barriers, geological history, and biotic interactions by following a suitable approach to deal with autocorrelation [42].

### Visualizing areas at risk from hunting

The advantage of accumulated favorability as a surrogate of diversity, compared to just summing species presences as employed in other studies [61], is that favorability is positively correlated with the abundance of a species at a given site [62], [63]. This approach has been used before by Estrada et al. [38] in which the accumulated favorability was used as part of a fuzzy set method for detecting diversity hot spots. Moreover, basing hot spots on favorability models have further allowed us to extrapolate observed patterns in this study to future human and climate scenarios. These will be published in a subsequent paper.

We delimited geographical clusters of taxa in the Central African region capable of sustaining variable levels of hunting extraction by singling out discrete weak spots or places of especial hunting vulnerability for wildlife, but also areas of high potential sustainability, such as strong spots. This approach is novel and can become a more realistic method for understanding where conservation efforts should be targeted. Hitherto, most spatial analyses of biodiversity in the Congo Basin have concentrated on the selection of conservation landscapes based on expert-driven assessments of the region's biological importance e.g. by the Congo Basin Forest Partnership [23]. Our approach goes beyond defining areas on the basis of species richness alone, focusing on what is the main form of human disturbance affecting many mammal species, hunting, and especially commercial hunting [64]–[67]. Moreover, areas that are considered completely irreplaceable for the conservation of African mammals are positively correlated with high human population density [68] and by association will be areas of highest hunting pressure.

Hot spots detected in our analysis coincide with areas of highest mammal richness in Africa as described by Rondinini et al. [61]. However, here we have gone a step further by applying fuzzy logic to turn a surrogate of diversity into a measurement of how sustainable this diversity is under hunting pressure. By weighting each species' favorability with an index of hunting sustainability (*PHS*, which, in fact, is a degree of membership into the fuzzy set of sustainable species), while keeping all species within the analysis, we have extracted information on how much the existing diversity at a location is subject to sustainable extraction (*SAF_j_*). Strong spots, the areas with highest *SAF_j_* values, are thus interpreted as a qualified hot spot for hunting. In contrast, by weighting species favorability values with the complementary fuzzy set of unsustainable species (1−*PHS*), we determine how much of the existing diversity at a location is unsustainable if subjected to hunting (*UAF_j_*). Weak spots point out areas where special policies should be implemented in order to protect species from overexploitation. Strong spots and weak spots can, nonetheless, overlap under some circumstances; these areas represent hot spots in which hunting is highly sustainable, on condition that only taxa with high *PHS* are the main quarry.

### Concluding remarks

A crucial part of the global policy agenda is the search for methods to understand the links between natural resources, economic activity and human well-being [69]. Among the priority issues is the attainment of hunting sustainability at a global scale [70]. This remains a key challenge because achieving equilibrium between hunter and quarry requires knowledge of the behavior, ecology and demography of the target species, but also of the economic costs and benefits, and institutional frameworks regulating animal harvests.

Similar to hot spots, weak spots — in spite of being qualified according to hunting sustainability of species — are nonetheless determined by high diversity. Rarity affected our analyses, but spatially restricted distributions often occupy areas with low overlap with other species. A clear example of this effect is the case of the Cross-Sanaga coastal forest region between Nigeria and Cameroon where high numbers of endemic species overlap, though not enough to be included within a weak spot (Fig. 4e). Thus, clusters of taxa with highly restricted distributions will require conservation policies that complement those targeting weak spots. These policies could be based on β-diversity, by focusing on enforcing representativeness of all species at risk of overhunting within the protected area network in Central Africa [71]; indeed, our main goal. Furthermore, our weak spots overlap with areas of high latent extinction risk for the Congo Basin shown in Cardillo et al. [55].

Additional analyses are needed to ascertain how weak and strong spots identified in our study are linked to anthropogenic pressures other than hunting, and how these may change in response to climate change. Moreover, how hunted mammal diversity is associated to human nutrition may advance our understanding of the importance of wild meat in the food security of forest inhabitants [8]. These topics are contemplated in subsequent papers.

## Supporting Information

File S1 **This file contains supporting appendices for this article.** Appendix S1, List of species included in the study, listed according to potential hunting sustainability (*PHS*). Appendix S2, Predictor variables (and primary sources) used to construct favorability models for all species in our study. Appendix S3, Detailed description of predictor variable design. Appendix S4, Number and percentage of taxa (species and subspecies) included in the five sustainability categories defined by potential hunting sustainability (*PHS*).
(DOC)
