# Supplementary material for: Integrating Sustainable Hunting in Biodiversity Protection in Central Africa: Hot Spots, Weak Spots, and Strong Spots
Source: PLoS One. 2014 Nov 5;9(11):e112367. doi: 10.1371/journal.pone.0112367 (PMC4221474; doi:10.1371/journal.pone.0112367)
Supplement: File S1 — This file contains supporting appendices for this article. Appendix S1, List of species included in the study, listed according to potential hunting sustainability (PHS). Appendix S2, Predictor variables (and primary sources) used to construct favorability models for all species in our study. Appendix S3, Detailed description of predictor variable design. Appendix S4, Number and percentage of taxa (species and subspecies) included in the five sustainability categories defined by potential hunting sustainability (PHS). (DOC) [file pone.0112367.s001.doc]

**Appendix S1**. **List of species included in the study, listed according to potential hunting sustainability (*PHS*):** *CS*: category of sustainability (sustainability increases from 1 to 5); *LogD*: decimal logarithm of density; *HB*: habitat breadth; *R*: rarity; *VS*: Vulnerability status. The black line indicates category limits; - : No data. Numbers in bold indicate threatened species and subspecies [*VS*/*VS*_max_ < 0.75, including vulnerable, endangered and critically endangered taxa] from classes 3, 4 and 5 whose distributions lay outside the limits of strong spots, and those in classes 1, 2 and 3 whose distributions were outside weak spot limits; most of these taxa are highly scored for rarity [(1-*R*)/(1-*R*)_max_ < 0.01]. Species and subspecies whose populations occur only in rainforests have an *HB*/*HB*_max_ = 0.14.

| **Permissive weighting** | | | | | | | | | |
| --- | --- | --- | --- | --- | --- | --- | --- | --- | --- |
| **Order** | **Species** | | ***PHS*** | **CS** | ***LogD*** | ***HB*/*HB*_max_** | | **(1-*R*)/(1-*R*)_max_** | ***VS*/*VS*_max_** |
| Hyracoidea | *Procavia capensis* | | 1.0000 | 5 | 3.44 | 0.43 | | 0.57 | 1.00 |
| Rodentia | *Funisciurus congicus* | | 0.8722 | 5 | 3.00 | 0.14 | | 0.07 | 1.00 |
| Rodentia | *Myosciurus pumilio* | | 0.8698 | 5 | 2.99 | 0.14 | | 0.01 | 1.00 |
| Rodentia | *Paraxerus alexandri* | | 0.7630 | 5 | 2.63 | 0.14 | | 0.01 | 1.00 |
| Primates | *Galagoides demidovii* | | 0.7410 | 5 | 2.55 | 0.43 | | 0.17 | 1.00 |
| Rodentia | *Paraxerus boehmi* | | 0.7322 | 5 | 2.52 | 0.29 | | 0.04 | 1.00 |
| Primates | *Galagoides thomasi* | | 0.7098 | 5 | 2.45 | 0.29 | | 0.18 | 1.00 |
| Rodentia | *Paraxerus poensis* | | 0.6988 | 5 | 2.41 | 0.29 | | 0.06 | 1.00 |
| Rodentia | *Funisciurus isabella* | | 0.6904 | 5 | 2.38 | 0.14 | | 0.02 | 1.00 |
| Rodentia | *Funisciurus lemniscatus* | | 0.6664 | 5 | 2.30 | 0.14 | | 0.02 | 1.00 |
| Primates | *Galago moholi* | | 0.6489 | 5 | 2.24 | 0.57 | | 0.17 | 1.00 |
| Primates | *Galago matschiei* | | 0.6280 | 5 | 2.17 | 0.14 | | 0.00 | 1.00 |
| Rodentia | *Paraxerus cepapi* | | 0.6232 | 5 | 2.15 | 0.14 | | 0.11 | 1.00 |
| Rodentia | *Funisciurus anerythrus* | | 0.6229 | 5 | 2.15 | 0.14 | | 0.09 | 1.00 |
| Primates | *Arctocebus aureus* | | 0.6185 | 5 | 2.13 | 0.14 | | 0.03 | 1.00 |
| Rodentia | *Cricetomys emini* | | 0.6174 | 5 | 2.13 | 0.14 | | 0.15 | 1.00 |
| Rodentia | *Funisciurus pyrropus* | | 0.6149 | 5 | 2.12 | 0.14 | | 0.08 | 1.00 |
| Rodentia | *Heliosciurus gambianus* | | 0.6130 | 5 | 2.12 | 0.29 | | 0.18 | 1.00 |
| Rodentia | *Funisciurus leucogenys* | | 0.6123 | 5 | 2.11 | 0.29 | | 0.02 | 1.00 |
| Primates | *Sciurocheirus gabonensis* | | 0.6086 | 5 | 2.10 | 0.14 | | 0.02 | 1.00 |
| Rodentia | *Funisciurus carruthersi* | | 0.6027 | 5 | 2.08 | 0.14 | | 0.00 | 1.00 |
| Primates | *Euoticus pallidus talboti* | | 0.6026 | 5 | 2.08 | 0.14 | | 0.00 | 1.00 |
| Carnivora | *Helogale parvula* | | 0.6011 | 5 | 2.07 | 0.29 | | 0.27 | 1.00 |
| Rodentia | *Heliosciurus ruwenzorii* | | 0.5980 | 5 | 2.06 | 0.43 | | 0.01 | 1.00 |
| Carnivora | *Poecilogale albinucha* | | 0.5927 | 5 | 2.05 | 0.57 | | 0.22 | 1.00 |
| Primates | *Cercopithecus ascanius ascanius* | | 0.5876 | 5 | 2.03 | 0.29 | | 0.02 | 1.00 |
| Primates | *Cercopithecus ascanius katangae* | | 0.5876 | 5 | 2.03 | 0.29 | | 0.02 | 1.00 |
| Primates | *Cercopithecus ascanius schmidti* | | 0.5876 | 5 | 2.03 | 0.29 | | 0.04 | 1.00 |
| Primates | *Cercopithecus ascanius whitesidei* | | 0.5876 | 5 | 2.03 | 0.29 | | 0.02 | 1.00 |
| Rodentia | *Heliosciurus rufobrachium* | | 0.5854 | 5 | 2.02 | 0.29 | | 0.11 | 1.00 |
| Primates | *Colobus guereza occidentalis* | | 0.5795 | 5 | 2.00 | 0.29 | | 0.08 | 1.00 |
| Primates | *Sciurocheirus alleni cameronensis* | | 0.5793 | 5 | 2.00 | 0.29 | | 0.00 | 1.00 |
| Primates | *Galago senegalensis* | | 0.5669 | 5 | 1.96 | 0.57 | | 0.33 | 1.00 |
| Primates | *Otolemur crassicaudatus* | | 0.5644 | 5 | 1.95 | 0.29 | | 0.19 | 1.00 |
| Rodentia | *Anomalurus beecrofti* | | 0.5512 | 5 | 1.90 | 0.29 | | 0.13 | 1.00 |
| Carnivora | *Herpestes sanguineus* | | 0.5458 | 5 | 1.89 | 1.00 | | 0.65 | 1.00 |
| Rodentia | *Epixerus ebii* | | 0.5434 | 5 | 1.88 | 0.14 | | 0.02 | 1.00 |
| Primates | *Cercopithecus mitis doggeti* | | 0.5363 | 5 | 1.85 | 0.14 | | 0.00 | 1.00 |
| Primates | *Cercopithecus mitis stuhlmanni* | | 0.5363 | 5 | 1.85 | 0.14 | | 0.02 | 1.00 |
| Rodentia | *Xerus erythropus* | | 0.5298 | 5 | 1.83 | 0.86 | | 0.34 | 1.00 |
| Rodentia | *Protoxerus stangeri* | | 0.5255 | 5 | 1.82 | 0.29 | | 0.13 | 1.00 |
| Afrosoricida | *Potamogale velox* | | 0.5197 | 4 | 1.80 | 0.14 | | 0.15 | 1.00 |
| Afrosoricida | *Micropotamogale ruwenzorii* | | 0.5163 | 4 | 2.38 | 0.14 | | 0.00 | 0.75 |
| Rodentia | *Atherurus africanus* | | 0.5055 | 4 | 1.75 | 0.29 | | 0.14 | 1.00 |
| Carnivora | *Ictonyx striatus* | | 0.5020 | 4 | 1.74 | 0.71 | | 0.60 | 1.00 |
| Rodentia | *Anomalurus derbianus* | | 0.4946 | 4 | 1.71 | 0.29 | | 0.22 | 1.00 |
| Carnivora | *Crossarchus platycephalus* | | 0.4730 | 4 | 1.64 | 0.57 | | 0.02 | 1.00 |
| Primates | *Miopithecus talapoin* | | 0.4721 | 4 | 1.63 | 0.14 | | 0.02 | 1.00 |
| Primates | *Miopithecus ogouensis* | | 0.4615 | 4 | 1.60 | 0.14 | | 0.02 | 1.00 |
| Rodentia | *Cricetomys gambianus* | | 0.4606 | 4 | 1.59 | 0.29 | | 0.33 | 1.00 |
| Cetartiodactyla | *Philantomba monticola* | | 0.4527 | 4 | 1.57 | 0.43 | | 0.25 | 1.00 |
| Carnivora | *Genetta thierryi* | | 0.4514 | 4 | 1.56 | 0.29 | | 0.10 | 1.00 |
| Carnivora | *Crossarchus alexandri* | | 0.4448 | 4 | 1.54 | 0.14 | | 0.06 | 1.00 |
| Carnivora | *Bdeogale crassicauda* | | 0.4323 | 4 | 1.50 | 0.14 | | 0.06 | 1.00 |
| Primates | *Cercopithecus neglectus* | | 0.4262 | 4 | 1.48 | 0.29 | | 0.09 | 1.00 |
| Carnivora | *Genetta maculata* | | 0.4209 | 4 | 1.46 | 0.71 | | 0.50 | 1.00 |
| Primates | *Cercopithecus mitis heymansi* | | 0.4006 | 4 | 1.85 | 0.14 | | 0.00 | 0.75 |
| Lagomorpha | *Poelagus marjorita* | | 0.3979 | 4 | 1.38 | 0.29 | | 0.01 | 1.00 |
| Hyracoidea | *Dendrohyrax arboreus* | | 0.3959 | 4 | 1.37 | 0.29 | | 0.10 | 1.00 |
| Primates | *Cercopithecus nictitans nictitans* | | 0.3950 | 4 | 1.37 | 0.29 | | 0.04 | 1.00 |
| Pholidota | *Phataginus tetradactyla* | | 0.3884 | 4 | 1.35 | 0.43 | | 0.11 | 1.00 |
| Primates | *Chlorocebus tantalus* | | 0.3879 | 4 | 1.35 | 0.29 | | 0.16 | 1.00 |
| Primates | *Cercopithecus cephus* | | 0.3869 | 4 | 1.34 | 0.14 | | 0.03 | 1.00 |
| Carnivora | *Xenogale naso* | | 0.3847 | 4 | 1.34 | 0.29 | | 0.10 | 1.00 |
| Carnivora | *Herpestes ichneumon* | | 0.3823 | 4 | 1.33 | 0.43 | | 0.55 | 1.00 |
| Hyracoidea | *Dendrohyrax dorsalis* | | 0.3766 | 4 | 1.31 | 0.43 | | 0.12 | 1.00 |
| Primates | *Cercopithecus pogonias grayi* | | 0.3748 | 4 | 1.30 | 0.14 | | 0.03 | 1.00 |
| Primates | *Cercopithecus pogonias nigripes* | | 0.3748 | 4 | 1.30 | 0.14 | | 0.01 | 1.00 |
| Primates | *Cercopithecus wolfi pyrogaster* | | 0.3743 | 4 | 1.30 | 0.14 | | 0.00 | 1.00 |
| Primates | *Cercopithecus wolfi wolfi* | | 0.3743 | 4 | 1.30 | 0.14 | | 0.02 | 1.00 |
| Primates | *Cercopithecus denti* | | 0.3678 | 4 | 1.28 | 0.14 | | 0.02 | 1.00 |
| Rodentia | *Thryonomys swinderianus* | | 0.3616 | 4 | 1.26 | 0.43 | | 0.29 | 1.00 |
| Carnivora | *Mungos mungo* | | 0.3610 | 4 | 1.25 | 0.29 | | 0.41 | 1.00 |
| Primates | *Lophocebus aterrimus* | | 0.3600 | 4 | 1.67 | 0.14 | | 0.02 | 0.75 |
| Primates | *Euoticus elegantulus* | | 0.3573 | 4 | 1.24 | 0.14 | | 0.03 | 1.00 |
| Primates | *Chlorocebus pygerythrus* | | 0.3525 | 4 | 1.23 | 0.43 | | 0.18 | 1.00 |
| Cetartiodactyla. | *Cephalophus callipygus* | | 0.3461 | 4 | 1.20 | 0.14 | | 0.03 | 1.00 |
| Primates | *Lophocebus albigena* | | 0.3450 | 4 | 1.20 | 0.29 | | 0.03 | 1.00 |
| Primates | *Lophocebus johnstoni* | | 0.3450 | 4 | 1.20 | 0.29 | | 0.03 | 1.00 |
| Primates | *Lophocebus osmani* | | 0.3450 | 4 | 1.20 | 0.29 | | 0.00 | 1.00 |
| Primates | *Lophocebus ugandae* | | 0.3450 | 4 | 1.20 | 0.29 | | 0.00 | 1.00 |
| Primates | *Chlorocebus cynosuros* | | 0.3443 | 4 | 1.20 | 0.29 | | 0.12 | 1.00 |
| Primates | *Allenopithecus nigroviridis* | | 0.3405 | 4 | 1.18 | 0.14 | | 0.02 | 1.00 |
| Primates | *Procolobus rufomitratus parmentieri* | | 0.3390 | 4 | 1.18 | 0.29 | | 0.00 | 1.00 |
| Carnivora | *Hydrictix maculicollis* | | 0.3360 | 4 | 1.17 | 0.43 | | 0.40 | 1.00 |
| Primates | *Procolobus rufomitratus oustaleti* | | 0.3303 | 4 | 1.15 | 0.29 | | 0.02 | 1.00 |
| Primates | *Cercopithecus mona* | | 0.3292 | 4 | 1.15 | 0.29 | | 0.02 | 1.00 |
| Cetartiodactyla | *Cephalophus ogilbyi crusalbum* | | 0.3292 | 4 | 1.15 | 0.29 | | 0.01 | 1.00 |
| Primates | *Procolobus rufomitratus lulindicus* | | 0.3210 | 4 | 1.12 | 0.29 | | 0.00 | 1.00 |
| Primates | *Procolobus rufomitratus langi* | | 0.3109 | 3 | 1.08 | 0.29 | | 0.00 | 1.00 |
| Primates | *Papio anubis* | | 0.3074 | 3 | 1.07 | 0.43 | | 0.31 | 1.00 |
| Primates | *Cercocebus agilis* | | 0.3052 | 3 | 1.06 | 0.14 | | 0.04 | 1.00 |
| Primates | *Procolobus rufomitratus foai* | | 0.2999 | 3 | 1.05 | 0.29 | | 0.00 | 1.00 |
| Pholidota | *Phataginus tricuspis* | | 0.2976 | 3 | 1.38 | 0.57 | | 0.23 | 0.75 |
| Carnivora | *Bdeogale jacksoni* | | 0.2970 | 3 | 1.38 | 0.29 | | 0.00 | 0.75 |
| Primates | *Cercopithecus erythrotis camerunensis* | | 0.2905 | 3 | 2.03 | 0.14 | | 0.00 | **0.50** |
| Primates | *Cercopithecus erythrotis erythrotis* | | 0.2905 | 3 | 2.03 | 0.14 | | 0.00 | **0.50** |
| Primates | *Procolobus rufomitratus ellioti* | | 0.2880 | 3 | 1.01 | 0.29 | | 0.00 | 1.00 |
| Primates | *Colobus angolensis angolensis* | | 0.2849 | 3 | 1.00 | 0.29 | | 0.07 | 1.00 |
| Primates | *Colobus angolensis cottoni* | | 0.2849 | 3 | 1.00 | 0.29 | | 0.01 | 1.00 |
| Carnivora | *Mellivora capensis* | | 0.2848 | 3 | 0.99 | 1.00 | | 1.00 | 1.00 |
| Primates | *Perodicticus potto potto* | | 0.2794 | 2 | 0.98 | 0.29 | | 0.02 | 1.00 |
| Primates | *Perodicticus potto ibeanus* | | 0.2794 | 2 | 0.98 | 0.29 | | 0.03 | 1.00 |
| Primates | *Perodicticus potto edwardsi* | | 0.2794 | 2 | 0.98 | 0.29 | | 0.09 | 1.00 |
| Primates | *Cercopithecus wolfi elegans* | | 0.2791 | 2 | 1.30 | 0.14 | | 0.00 | 0.75 |
| Primates | *Cercocebus chrysogaster* | | 0.2734 | 2 | 0.96 | 0.14 | | 0.01 | 1.00 |
| Cetartiodactyla | *Hyemoschus aquaticus* | | 0.2690 | 2 | 0.94 | 0.14 | | 0.10 | 1.00 |
| Primates | *Procolobus rufomitratus tholloni* | | 0.2645 | 2 | 1.23 | 0.29 | | 0.02 | 0.75 |
| Carnivora | *Caracal caracal* | | 0.2608 | 2 | 0.91 | 0.71 | | 0.63 | 1.00 |
| Carnivora | *Leptailurus serval* | | 0.2606 | 2 | 0.91 | 0.86 | | 0.49 | 1.00 |
| Rodentia | *Hystrix cristata* | | 0.2514 | 2 | 0.88 | 0.86 | | 0.20 | 1.00 |
| Rodentia | *Anomalurus pusillus* | | 0.2449 | 2 | 0.86 | 0.14 | | 0.02 | 1.00 |
| Primates | *Arctocebus calabarensis* | | 0.2446 | 2 | 0.86 | 0.29 | | 0.01 | 1.00 |
| Rodentia | *Hystrix africaeaustralis* | | 0.2425 | 2 | 0.85 | 0.57 | | 0.30 | 1.00 |
| Primates | *Papio cynocephalus* | | 0.2384 | 2 | 0.84 | 0.43 | | 0.17 | 1.00 |
| Cetartiodactyla | *Syncerus caffer brachyceros* | | 0.2381 | 2 | 0.84 | 0.43 | | 0.04 | 1.00 |
| Cetartiodactyla | *Syncerus caffer nanus* | | 0.2381 | 2 | 0.84 | 0.43 | | 0.10 | 1.00 |
| Cetartiodactyla | *Syncerus caffer caffer* | | 0.2381 | 2 | 0.84 | 0.43 | | 0.14 | 1.00 |
| Cetartiodactyla | *Syncerus caffer aequinoctialis* | | 0.2381 | 2 | 0.84 | 0.43 | | 0.05 | 1.00 |
| Cetartiodactyla | *Cephalophus weynsi* | | 0.2306 | 2 | 0.81 | 0.14 | | 0.06 | 1.00 |
| Primates | *Cercocebus torquatus* | | 0.2299 | 2 | 1.61 | 0.43 | | 0.01 | 0.50 |
| Carnivora | *Nandinia binotata* | | 0.2212 | 2 | 0.78 | 0.57 | | 0.24 | 1.00 |
| Primates | *Colobus angolensis cordeiri* | | 0.2121 | 2 | 1.00 | 0.29 | | 0.00 | 0.75 |
| Carnivora | *Genetta cristata* | | 0.2093 | 2 | 1.47 | 0.14 | | 0.00 | **0.50** |
| Carnivora | *Aonyx congicus* | | 0.2049 | 2 | 0.72 | 0.29 | | 0.10 | 1.00 |
| Primates | *Colobus satanas anthracinus* | | 0.2044 | 2 | 1.44 | 0.14 | | 0.01 | 0.50 |
| Cetartiodactyla | *Cephalophus leucogaster* | | 0.2007 | 2 | 0.71 | 0.14 | | 0.05 | 1.00 |
| Primates | *Cercopithecus nictitans martini* | | 0.1943 | 2 | 1.37 | 0.29 | | 0.01 | **0.50** |
| Cetartiodactyla | *Cephalophus dorsalis* | | 0.1930 | 2 | 0.68 | 0.29 | | 0.12 | 1.00 |
| Primates | *Mandrillus sphinx* | | 0.1912 | 2 | 1.35 | 0.14 | | 0.01 | 0.50 |
| Carnivora | *Ichneumia albicauda* | | 0.1904 | 2 | 0.67 | 0.43 | | 0.53 | 1.00 |
| Rodentia | *Funisciurus bayonii* | | 0.1870 | 2 | 2.31 | 0.29 | | 0.02 | - |
| Primates | *Cercopithecus sclateri* | | 0.1866 | 2 | 1.32 | 0.14 | | 0.00 | **0.50** |
| Primates | *Cercopithecus pogonias pogonias* | | 0.1842 | 2 | 1.30 | 0.14 | | 0.00 | 0.50 |
| Cetartiodactyla | *Cephalophus rufilatus* | | 0.1754 | 2 | 0.62 | 0.57 | | 0.11 | 1.00 |
| Primates | *Cercopithecus hamlyni* | | 0.1689 | 2 | 1.20 | 0.14 | | 0.01 | 0.50 |
| Primates | *Allochrocebus lhoesti* | | 0.1666 | 2 | 1.18 | 0.29 | | 0.01 | 0.50 |
| Carnivora | *Civettictis civetta* | | 0.1633 | 2 | 0.58 | 0.43 | | 0.60 | 1.00 |
| Primates | *Cercopithecus ascanius atrinasus* | | 0.1633 | 2 | 2.03 | 0.29 | | 0.00 | - |
| Cetartiodactyla | *Cephalophus ogilbyi ogilbyi* | | 0.1614 | 2 | 1.15 | 0.29 | | 0.00 | **0.50** |
| Primates | *Colobus guereza matschiei* | | 0.1609 | 2 | 2.00 | 0.29 | | 0.00 |  |
| Primates | *Allochrocebus solatus* | | 0.1609 | 2 | 1.14 | 0.14 | | 0.00 | **0.50** |
| Primates | *Sciurocheirus alleni alleni* | | 0.1605 | 2 | 2.00 | 0.29 | | 0.00 | **0.25** |
| Carnivora | *Dologale dybowskii* | | 0.1604 | 2 | 1.99 | 0.29 | | 0.02 | - |
| Primates | *Cercopithecus lomamiensis* | | 0.1601 | 2 | 1.14 | 0.14 | | 0.00 | **0.50** |
| Primates | *Lophocebus opdenboschi* | | 0.1532 | 2 | 1.09 | 0.14 | | 0.01 | **0.50** |
| Carnivora | *Atilax paludinosus* | | 0.1529 | 2 | 0.54 | 0.14 | | 0.55 | 1.00 |
| Primates | *Euoticus pallidus pallidus* | | 0.1458 | 2 | 2.08 | 0.14 | | 0.00 | **0.25** |
| Tubulidentata | *Orycteropus afer* | | 0.1440 | 2 | 0.51 | 0.71 | | 0.75 | 1.00 |
| Proboscidea | *Loxodonta africana* | | 0.1421 | 2 | 0.89 | 0.57 | | 0.10 | 0.50 |
| Primates | *Colobus angolensis ruwenzorii* | | 0.1392 | 2 | 1.00 | 0.29 | | 0.00 | 0.50 |
| Pholidota | *Smutsia gigantea* | | 0.1350 | 2 | 0.64 | 0.57 | | 0.13 | 0.75 |
| Cetartiodactyla | *Cephalophus nigrifrons* | | 0.1333 | 2 | 0.48 | 0.14 | | 0.12 | 1.00 |
| Carnivora | *Genetta genetta* | | 0.1281 | 2 | 0.46 | 0.71 | | 0.53 | 1.00 |
| Cetartiodactyla | *Phacochoerus africanus* | | 0.1198 | 2 | 0.43 | 0.43 | | 0.43 | 1.00 |
| Cetartiodactyla | *Tragelaphus spekii* | | 0.1180 | 2 | 0.42 | 0.57 | | 0.17 | 1.00 |
| Cetartiodactyla | *Tragelaphus scriptus* | | 0.1111 | 2 | 0.40 | 0.71 | | 0.47 | 1.00 |
| Cetartiodactyla | *Hylochoerus meinertzhageni* | | 0.1101 | 1 | 0.40 | 0.43 | | 0.08 | 1.00 |
| Cetartiodactyla | *Neotragus batesi batesi* | | 0.1101 | 1 | 0.40 | 0.14 | | 0.02 | 1.00 |
| Cetartiodactyla | *Neotragus batesi harrisoni* | | 0.1101 | 1 | 0.40 | 0.14 | | 0.01 | 1.00 |
| Cetartiodactyla | *Hippopotamus amphibius* | | 0.1060 | 1 | 0.77 | 0.43 | | 0.07 | 0.50 |
| Primates | *Colobus satanas satanas* | | 0.0990 | 1 | 1.44 | 0.14 | | 0.00 | **0.25** |
| Primates | *Procolobus rufomitratus tephrosceles* | | 0.0946 | 1 | 1.21 | 0.29 | | 0.00 | **0.25** |
| Carnivora | *Genetta servalina* | | 0.0938 | 1 | 0.34 | 0.14 | | 0.12 | 1.00 |
| Primates | *Pan paniscus* | | 0.0933 | 1 | 0.48 | 0.71 | | 0.02 | **0.25** |
| Rodentia | *Funisciurus duchaillui* | | 0.0849 | 1 | 2.18 | 0.14 | | 0.00 | - |
| Rodentia | *Paraxerus cooperi* | | 0.0819 | 1 | 2.11 | 0.14 | | 0.00 | - |
| Carnivora | *Poiana richardsonii* | | 0.0817 | 1 | 0.30 | 0.14 | | 0.08 | 1.00 |
| Primates | *Allochrocebus preussi ins* | | 0.0815 | 1 | 1.20 | 0.14 | | 0.00 | **0.25** |
| Primates | *Allochrocebus preussi pre* | | 0.0815 | 1 | 1.20 | 0.14 | | 0.00 | **0.25** |
| Cetartiodactyla | *Hippotragus equinus* | | 0.0791 | 1 | 0.29 | 0.29 | | 0.22 | 1.00 |
| Primates | *Colobus angolensis prigoginei* | | 0.0768 | 1 | 1.00 | 0.29 | | 0.00 | **0.25** |
| Primates | *Cercopithecus mitis mitis* | | 0.0711 | 1 | 1.85 | 0.14 | | 0.00 | - |
| Primates | *Cercopithecus mitis schoutedeni* | | 0.0711 | 1 | 1.85 | 0.14 | | 0.00 | **0.00** |
| Carnivora | *Crossarchus ansorgei* | | 0.0681 | 1 | 1.78 | 0.14 | | 0.02 | - |
| Primates | *Procolobus pennantii pennantii* | | 0.0665 | 1 | 1.00 | 0.14 | | 0.00 | **0.25** |
| Primates | *Mandrillus leucophaeus leucophaeus* | | 0.0658 | 1 | 0.86 | 0.29 | | 0.00 | **0.25** |
| Primates | *Mandrillus leucophaeus poensis* | | 0.0658 | 1 | 0.86 | 0.29 | | 0.00 | **0.25** |
| Carnivora | *Genetta piscivora* | | 0.0568 | 1 | 1.51 | 0.14 | | 0.01 | - |
| Primates | *Cercopithecus dryas* | | 0.0500 | 1 | 1.35 | 0.14 | | 0.00 | **0.00** |
| Carnivora | *Genetta poensis* | | 0.0486 | 1 | 1.32 | 0.14 | | 0.01 | - |
| Perissodactyla | *Equus quagga* | | 0.0471 | 1 | 0.18 | 0.43 | | 0.09 | 1.00 |
| Cetartiodactyla | *Tragelaphus oryx* | | 0.0456 | 1 | 0.18 | 0.43 | | 0.26 | 1.00 |
| Cetartiodactyla | *Cephalophus silvicultor* | | 0.0451 | 1 | 0.18 | 0.43 | | 0.24 | 1.00 |
| Primates | *Procolobus preussi* | | 0.0377 | 1 | 1.06 | 0.14 | | 0.00 | **0.00** |
| Cetartiodactyla | *Potamochoerus larvatus* | | 0.0363 | 1 | 0.15 | 0.43 | | 0.23 | 1.00 |
| Carnivora | *Genetta victoriae* | | 0.0363 | 1 | 0.15 | 0.14 | | 0.02 | 1.00 |
| Carnivora | *Bdeogale nigripes* | | 0.0363 | 1 | 0.15 | 0.14 | | 0.08 | 1.00 |
| Primates | *Procolobus pennantii bouvieri* | | 0.0333 | 1 | 0.95 | 0.14 | | 0.00 | **0.00** |
| Carnivora | *Felis silvestris* | | 0.0327 | 1 | 0.13 | 0.57 | | 0.88 | 1.00 |
| Cetartiodactyla | *Tragelaphus derbianus* | | 0.0326 | 1 | 0.13 | 0.29 | | 0.01 | 1.00 |
| Carnivora | *Aonyx capensis* | | 0.0317 | 1 | 0.13 | 0.43 | | 0.46 | 1.00 |
| Primates | *Pan troglodytes schweinfurthii* | | 0.0287 | 1 | 0.42 | 0.29 | | 0.04 | 0.25 |
| Primates | *Pan troglodytes troglodytes* | | 0.0287 | 1 | 0.42 | 0.29 | | 0.03 | 0.25 |
| Primates | *Pan troglodytes ellioti* | | 0.0287 | 1 | 0.42 | 0.29 | | 0.01 | **0.25** |
| Cetartiodactyla | *Tragelaphus eurycerus* | | 0.0256 | 1 | 0.15 | 0.57 | | 0.09 | 0.75 |
| Cetartiodactyla | *Potamochoerus porcus* | | 0.0199 | 1 | 0.09 | 0.43 | | 0.20 | 1.00 |
| Proboscidea | *Loxodonta cyclotis* | | 0.0193 | 1 | 0.18 | 0.43 | | 0.03 | 0.50 |
| Carnivora | *Canis adustus* | | 0.0188 | 1 | 0.09 | 0.57 | | 0.42 | 1.00 |
| Primates | *Gorilla beringei graueri* | | 0.0170 | 1 | 0.32 | 0.14 | | 0.00 | 0.25 |
| Cetartiodactyla | *Okapia johnstoni* | | 0.0159 | 1 | 0.31 | 0.14 | | 0.01 | 0.25 |
| Carnivora | *Profelis aurata* | | 0.0109 | 1 | 0.08 | 0.57 | | 0.16 | 0.75 |
| Carnivora | *Crocuta crocuta* | | 0.0080 | 1 | 0.05 | 1.00 | | 0.57 | 1.00 |
| Primates | *Gorilla beringei beringei* | | 0.0070 | 1 | 0.32 | 0.14 | | 0.00 | **0.00** |
| Primates | *Gorilla gorilla diehli* | | 0.0020 | 1 | 0.20 | 0.14 | | 0.00 | **0.00** |
| Primates | *Gorilla gorilla gorilla* | | 0.0020 | 1 | 0.20 | 0.14 | | 0.03 | 0.00 |
| Perissodactyla | *Diceros bicornis* | | 0.0011 | 1 | 0.05 | 0.57 | | 0.20 | **0.00** |
| Carnivora | *Panthera pardus* | | 0.0000 | 1 | 0.03 | 0.57 | | 0.64 | 0.75 |
| **Restrictive weighting** | | | | | | | | | |
| **Order** | ***Species*** | | ***PHS*** | **CS** | ***LogD*** | ***HB*/*HB_ma_*_x_** | | **(1-*R*)/(1-*R)_max_*** | ***VS*/*VS_max_*** |
| Hyracoidea | | *Procavia capensis* | 1.0000 | 5 | 3.44 | 0.43 | 0.57 | | 1.00 |
| Carnivora | | *Herpestes sanguineus* | 0.8352 | 5 | 1.89 | 1.00 | 0.65 | | 1.00 |
| Carnivora | | *Ictonyx striatus* | 0.7062 | 5 | 1.74 | 0.71 | 0.60 | | 1.00 |
| Carnivora | | *Mellivora capensis* | 0.6753 | 5 | 0.99 | 1.00 | 1.00 | | 1.00 |
| Carnivora | | *Genetta maculata* | 0.4973 | 5 | 1.46 | 0.71 | 0.50 | | 1.00 |
| Primates | | *Galago senegalensis* | 0.4353 | 5 | 1.96 | 0.57 | 0.33 | | 1.00 |
| Rodentia | | *Xerus erythropus* | 0.4279 | 5 | 1.83 | 0.86 | 0.34 | | 1.00 |
| Carnivora | | *Caracal caracal* | 0.3888 | 5 | 0.91 | 0.71 | 0.63 | | 1.00 |
| Carnivora | | *Herpestes ichneumon* | 0.3862 | 5 | 1.33 | 0.43 | 0.55 | | 1.00 |
| Carnivora | | *Helogale parvula* | 0.3804 | 5 | 2.07 | 0.29 | 0.27 | | 1.00 |
| Carnivora | | *Hydrictix maculicollis* | 0.3187 | 5 | 1.17 | 0.43 | 0.40 | | 1.00 |
| Rodentia | | *Cricetomys gambianus* | 0.3093 | 5 | 1.59 | 0.29 | 0.33 | | 1.00 |
| Carnivora | | *Poecilogale albinucha* | 0.3071 | 5 | 2.05 | 0.57 | 0.22 | | 1.00 |
| Carnivora | | *Leptailurus serval* | 0.3027 | 5 | 0.91 | 0.86 | 0.49 | | 1.00 |
| Primates | | *Galagoides demidovii* | 0.2975 | 5 | 2.55 | 0.43 | 0.17 | | 1.00 |
| Primates | | *Galagoides thomasi* | 0.2918 | 5 | 2.45 | 0.29 | 0.18 | | 1.00 |
| Cetartiodactyla | | *Philantomba monticola* | 0.2649 | 5 | 1.57 | 0.43 | 0.25 | | 1.00 |
| Rodentia | | *Heliosciurus gambianus* | 0.2643 | 5 | 2.12 | 0.29 | 0.18 | | 1.00 |
| Rodentia | | *Anomalurus derbianus* | 0.2609 | 5 | 1.71 | 0.29 | 0.22 | | 1.00 |
| Primates | | *Otolemur crassicaudatus* | 0.2529 | 5 | 1.95 | 0.29 | 0.19 | | 1.00 |
| Primates | | *Galago moholi* | 0.2519 | 5 | 2.24 | 0.57 | 0.17 | | 1.00 |
| Rodentia | | *Thryonomys swinderianus* | 0.2512 | 5 | 1.26 | 0.43 | 0.29 | | 1.00 |
| Tubulidentata | | *Orycteropus afer* | 0.2491 | 5 | 0.51 | 0.71 | 0.75 | | 1.00 |
| Carnivora | | *Mungos mungo* | 0.2434 | 5 | 1.25 | 0.29 | 0.41 | | 1.00 |
| Primates | | *Papio anubis* | 0.2224 | 5 | 1.07 | 0.43 | 0.31 | | 1.00 |
| Pholidota | | *Phataginus tricuspis* | 0.2177 | 5 | 1.38 | 0.57 | 0.23 | | 0.75 |
| Rodentia | | *Cricetomys emini* | 0.2066 | 5 | 2.13 | 0.14 | 0.15 | | 1.00 |
| Carnivora | | *Ichneumia albicauda* | 0.1955 | 5 | 0.67 | 0.43 | 0.53 | | 1.00 |
| Afrosoricida | | *Potamogale velox* | 0.1743 | 5 | 1.80 | 0.14 | 0.15 | | 1.00 |
| Rodentia | | *Hystrix africaeaustralis* | 0.1718 | 5 | 0.85 | 0.57 | 0.30 | | 1.00 |
| Carnivora | | *Civettictis civetta* | 0.1687 | 5 | 0.58 | 0.43 | 0.60 | | 1.00 |
| Carnivora | | *Genetta genetta* | 0.1660 | 5 | 0.46 | 0.71 | 0.53 | | 1.00 |
| Rodentia | | *Atherurus africanus* | 0.1642 | 4 | 1.75 | 0.29 | 0.14 | | 1.00 |
| Rodentia | | *Anomalurus beecrofti* | 0.1638 | 4 | 1.90 | 0.29 | 0.13 | | 1.00 |
| Rodentia | | *Protoxerus stangeri* | 0.1632 | 4 | 1.82 | 0.29 | 0.13 | | 1.00 |
| Rodentia | | *Heliosciurus rufobrachium* | 0.1573 | 4 | 2.02 | 0.29 | 0.11 | | 1.00 |
| Rodentia | | *Paraxerus cepapi* | 0.1534 | 4 | 2.15 | 0.14 | 0.11 | | 1.00 |
| Primates | | *Chlorocebus pygerythrus* | 0.1487 | 4 | 1.23 | 0.43 | 0.18 | | 1.00 |
| Primates | | *Chlorocebus tantalus* | 0.1424 | 4 | 1.35 | 0.29 | 0.16 | | 1.00 |
| Rodentia | | *Funisciurus congicus* | 0.1380 | 4 | 3.00 | 0.14 | 0.07 | | 1.00 |
| Rodentia | | *Funisciurus anerythrus* | 0.1299 | 4 | 2.15 | 0.14 | 0.09 | | 1.00 |
| Cetartiodactyla | | *Tragelaphus scriptus* | 0.1283 | 4 | 0.40 | 0.71 | 0.47 | | 1.00 |
| Cetartiodactyla | | *Phacochoerus africanus* | 0.1251 | 4 | 0.43 | 0.43 | 0.43 | | 1.00 |
| Carnivora | | *Nandinia binotata* | 0.1241 | 4 | 0.78 | 0.57 | 0.24 | | 1.00 |
| Rodentia | | *Hystrix cristata* | 0.1224 | 4 | 0.88 | 0.86 | 0.20 | | 1.00 |
| Rodentia | | *Funisciurus pyrropus* | 0.1211 | 4 | 2.12 | 0.14 | 0.08 | | 1.00 |
| Primates | | *Colobus guereza occidentalis* | 0.1084 | 4 | 2.00 | 0.29 | 0.08 | | 1.00 |
| Carnivora | | *Genetta thierryi* | 0.1071 | 4 | 1.56 | 0.29 | 0.10 | | 1.00 |
| Hyracoidea | | *Dendrohyrax dorsalis* | 0.1068 | 4 | 1.31 | 0.43 | 0.12 | | 1.00 |
| Rodentia | | *Paraxerus poensis* | 0.1003 | 4 | 2.41 | 0.29 | 0.06 | | 1.00 |
| Primates | | *Chlorocebus cynosuros* | 0.0979 | 4 | 1.20 | 0.29 | 0.12 | | 1.00 |
| Pholidota | | *Phataginus tetradactyla* | 0.0969 | 4 | 1.35 | 0.43 | 0.11 | | 1.00 |
| Primates | | *Papio cynocephalus* | 0.0951 | 4 | 0.84 | 0.43 | 0.17 | | 1.00 |
| Carnivora | | *Xenogale naso* | 0.0941 | 4 | 1.34 | 0.29 | 0.10 | | 1.00 |
| Primates | | *Cercopithecus neglectus* | 0.0897 | 3 | 1.48 | 0.29 | 0.09 | | 1.00 |
| Hyracoidea | | *Dendrohyrax arboreus* | 0.0891 | 3 | 1.37 | 0.29 | 0.10 | | 1.00 |
| Cetartiodactyla | | *Syncerus caffer caffer* | 0.0815 | 3 | 0.84 | 0.43 | 0.14 | | 1.00 |
| Rodentia | | *Paraxerus boehmi* | 0.0639 | 2 | 2.52 | 0.29 | 0.04 | | 1.00 |
| Primates | | *Perodicticus potto edwardsi* | 0.0616 | 2 | 0.98 | 0.29 | 0.09 | | 1.00 |
| Proboscidea | | *Loxodonta africana* | 0.0615 | 2 | 0.89 | 0.57 | 0.10 | | 0.50 |
| Primates | | *Cercopithecus ascanius schmidti* | 0.0613 | 2 | 2.03 | 0.29 | 0.04 | | 1.00 |
| Cetartiodactyla | | *Hyemoschus aquaticus* | 0.0611 | 2 | 0.94 | 0.14 | 0.10 | | 1.00 |
| Carnivora | | *Bdeogale crassicauda* | 0.0577 | 2 | 1.50 | 0.14 | 0.06 | | 1.00 |
| Carnivora | | *Crossarchus alexandri* | 0.0577 | 2 | 1.54 | 0.14 | 0.06 | | 1.00 |
| Pholidota | | *Smutsia gigantea* | 0.0561 | 2 | 0.64 | 0.57 | 0.13 | | 0.75 |
| Cetartiodactyla | | *Syncerus caffer nanus* | 0.0552 | 2 | 0.84 | 0.43 | 0.10 | | 1.00 |
| Cetartiodactyla | | *Cephalophus dorsalis* | 0.0536 | 2 | 0.68 | 0.29 | 0.12 | | 1.00 |
| Carnivora | | *Atilax paludinosus* | 0.0528 | 2 | 0.54 | 0.14 | 0.55 | | 1.00 |
| Carnivora | | *Felis silvestris* | 0.0518 | 2 | 0.13 | 0.57 | 0.88 | | 1.00 |
| Carnivora | | *Aonyx congicus* | 0.0511 | 2 | 0.72 | 0.29 | 0.10 | | 1.00 |
| Cetartiodactyla | | *Tragelaphus spekii* | 0.0495 | 2 | 0.42 | 0.57 | 0.17 | | 1.00 |
| Cetartiodactyla | | *Cephalophus rufilatus* | 0.0482 | 2 | 0.62 | 0.57 | 0.11 | | 1.00 |
| Primates | | *Colobus angolensis angolensis* | 0.0447 | 2 | 1.00 | 0.29 | 0.07 | | 1.00 |
| Cetartiodactyla | | *Hippotragus equinus* | 0.0432 | 2 | 0.29 | 0.29 | 0.22 | | 1.00 |
| Cetartiodactyla | | *Cephalophus nigrifrons* | 0.0404 | 2 | 0.48 | 0.14 | 0.12 | | 1.00 |
| Cetartiodactyla | | *Hippopotamus amphibius* | 0.0383 | 2 | 0.77 | 0.43 | 0.07 | | 0.50 |
| Carnivora | | *Aonyx capensis* | 0.0379 | 2 | 0.13 | 0.43 | 0.46 | | 1.00 |
| Primates | | *Arctocebus aureus* | 0.0378 | 2 | 2.13 | 0.14 | 0.03 | | 1.00 |
| Rodentia | | *Funisciurus lemniscatus* | 0.0372 | 2 | 2.30 | 0.14 | 0.02 | | 1.00 |
| Rodentia | | *Funisciurus isabella* | 0.0360 | 2 | 2.38 | 0.14 | 0.02 | | 1.00 |
| Primates | | *Cercopithecus nictitans nictitans* | 0.0351 | 2 | 1.37 | 0.29 | 0.04 | | 1.00 |
| Cetartiodactyla | | *Tragelaphus oryx* | 0.0319 | 2 | 0.18 | 0.43 | 0.26 | | 1.00 |
| Cetartiodactyla | | *Cephalophus weynsi* | 0.0319 | 2 | 0.81 | 0.14 | 0.06 | | 1.00 |
| Primates | | *Cercopithecus ascanius katangae* | 0.0318 | 2 | 2.03 | 0.29 | 0.02 | | 1.00 |
| Primates | | *Cercocebus agilis* | 0.0297 | 2 | 1.06 | 0.14 | 0.04 | | 1.00 |
| Primates | | *Sciurocheirus gabonensis* | 0.0296 | 2 | 2.10 | 0.14 | 0.02 | | 1.00 |
| Carnivora | | *Genetta servalina* | 0.0288 | 2 | 0.34 | 0.14 | 0.12 | | 1.00 |
| Cetartiodactyla | | *Cephalophus silvicultor* | 0.0288 | 2 | 0.18 | 0.43 | 0.24 | | 1.00 |
| Carnivora | | *Crossarchus ansorgei* | 0.0285 | 2 | 1.78 | 0.14 | 0.02 | | - |
| Cetartiodactyla | | *Syncerus caffer aequinoctialis* | 0.0284 | 2 | 0.84 | 0.43 | 0.05 | | 1.00 |
| Primates | | *Cercopithecus cephus* | 0.0277 | 2 | 1.34 | 0.14 | 0.03 | | 1.00 |
| Rodentia | | *Myosciurus pumilio* | 0.0275 | 2 | 2.99 | 0.14 | 0.01 | | 1.00 |
| Primates | | *Cercopithecus ascanius whitesidei* | 0.0270 | 2 | 2.03 | 0.29 | 0.02 | | 1.00 |
| Rodentia | | *Epixerus ebii* | 0.0269 | 2 | 1.88 | 0.14 | 0.02 | | 1.00 |
| Primates | | *Cercopithecus mitis stuhlmanni* | 0.0268 | 2 | 1.85 | 0.14 | 0.02 | | 1.00 |
| Primates | | *Euoticus elegantulus* | 0.0258 | 2 | 1.24 | 0.14 | 0.03 | | 1.00 |
| Carnivora | | *Dologale dybowskii* | 0.0256 | 2 | 1.99 | 0.29 | 0.02 | | - |
| Primates | | *Lophocebus aterrimus* | 0.0255 | 2 | 1.67 | 0.14 | 0.02 | | 0.75 |
| Primates | | *Cercopithecus pogonias grayi* | 0.0250 | 2 | 1.30 | 0.14 | 0.03 | | 1.00 |
| Carnivora | | *Canis adustus* | 0.0245 | 2 | 0.09 | 0.57 | 0.42 | | 1.00 |
| Cetartiodactyla | | *Cephalophus callipygus* | 0.0238 | 2 | 1.20 | 0.14 | 0.03 | | 1.00 |
| Rodentia | | *Funisciurus bayonii* | 0.0236 | 2 | 2.31 | 0.29 | 0.02 | | - |
| Cetartiodactyla | | *Cephalophus leucogaster* | 0.0234 | 2 | 0.71 | 0.14 | 0.05 | | 1.00 |
| Primates | | *Cercopithecus ascanius ascanius* | 0.0232 | 2 | 2.03 | 0.29 | 0.02 | | 1.00 |
| Primates | | *Perodicticus potto ibeanus* | 0.0231 | 2 | 0.98 | 0.29 | 0.03 | | 1.00 |
| Rodentia | | *Funisciurus leucogenys* | 0.0231 | 2 | 2.11 | 0.29 | 0.02 | | 1.00 |
| Cetartiodactyla | | *Potamochoerus larvatus* | 0.0228 | 2 | 0.15 | 0.43 | 0.23 | | 1.00 |
| Primates | | *Lophocebus albigena* | 0.0227 | 2 | 1.20 | 0.29 | 0.03 | | 1.00 |
| Cetartiodactyla | | *Hylochoerus meinertzhageni* | 0.0227 | 2 | 0.40 | 0.43 | 0.08 | | 1.00 |
| Primates | | *Lophocebus johnstoni* | 0.0227 | 2 | 1.20 | 0.29 | 0.03 | | 1.00 |
| Carnivora | | *Crossarchus platycephalus* | 0.0220 | 2 | 1.64 | 0.57 | 0.02 | | 1.00 |
| Cetartiodactyla | | *Syncerus caffer brachyceros* | 0.0218 | 2 | 0.84 | 0.43 | 0.04 | | 1.00 |
| Primates | | *Miopithecus ogouensis* | 0.0206 | 2 | 1.60 | 0.14 | 0.02 | | 1.00 |
| Carnivora | | *Crocuta crocuta* | 0.0189 | 2 | 0.05 | 1.00 | 0.57 | | 1.00 |
| Primates | | *Cercopithecus mona* | 0.0187 | 2 | 1.15 | 0.29 | 0.02 | | 1.00 |
| Rodentia | | *Paraxerus alexandri* | 0.0180 | 2 | 2.63 | 0.14 | 0.01 | | 1.00 |
| Primates | | *Procolobus rufomitratus oustaleti* | 0.0178 | 2 | 1.15 | 0.29 | 0.02 | | 1.00 |
| Primates | | *Cercopithecus wolfi wolfi* | 0.0171 | 2 | 1.30 | 0.14 | 0.02 | | 1.00 |
| Primates | | *Miopithecus talapoin* | 0.0169 | 2 | 1.63 | 0.14 | 0.02 | | 1.00 |
| Primates | | *Perodicticus potto potto* | 0.0165 | 2 | 0.98 | 0.29 | 0.02 | | 1.00 |
| Primates | | *Procolobus rufomitratus tholloni* | 0.0163 | 2 | 1.23 | 0.29 | 0.02 | | 0.75 |
| Carnivora | | *Poiana richardsonii* | 0.0161 | 2 | 0.30 | 0.14 | 0.08 | | 1.00 |
| Primates | | *Allenopithecus nigroviridis* | 0.0159 | 2 | 1.18 | 0.14 | 0.02 | | 1.00 |
| Primates | | *Cercopithecus denti* | 0.0138 | 2 | 1.28 | 0.14 | 0.02 | | 1.00 |
| Primates | | *Cercopithecus nictitans martini* | 0.0135 | 2 | 1.37 | 0.29 | 0.01 | | **0.50** |
| Rodentia | | *Anomalurus pusillus* | 0.0130 | 2 | 0.86 | 0.14 | 0.02 | | 1.00 |
| Primates | | *Colobus satanas anthracinus* | 0.0125 | 2 | 1.44 | 0.14 | 0.01 | | 0.50 |
| Primates | | *Cercocebus torquatus* | 0.0123 | 2 | 1.61 | 0.43 | 0.01 | | 0.50 |
| Cetartiodactyla | | *Potamochoerus porcus* | 0.0119 | 2 | 0.09 | 0.43 | 0.20 | | 1.00 |
| Primates | | *Mandrillus sphinx* | 0.0116 | 2 | 1.35 | 0.14 | 0.01 | | 0.50 |
| Carnivora | | *Panthera pardus* | 0.0114 | 2 | 0.03 | 0.57 | 0.64 | | 0.75 |
| Perissodactyla | | *Equus quagga* | 0.0112 | 2 | 0.18 | 0.43 | 0.09 | | 1.00 |
| Primates | | *Pan troglodytes schweinfurthii* | 0.0107 | 2 | 0.42 | 0.29 | 0.04 | | 0.25 |
| Carnivora | | *Genetta piscivora* | 0.0090 | 2 | 1.51 | 0.14 | 0.01 | | - |
| Carnivora | | *Profelis aurata* | 0.0086 | 2 | 0.08 | 0.57 | 0.16 | | 0.75 |
| Cetartiodactyla | | *Tragelaphus eurycerus* | 0.0086 | 2 | 0.15 | 0.57 | 0.09 | | 0.75 |
| Primates | | *Cercopithecus pogonias nigripes* | 0.0082 | 2 | 1.30 | 0.14 | 0.01 | | 1.00 |
| Carnivora | | *Bdeogale nigripes* | 0.0082 | 2 | 0.15 | 0.14 | 0.08 | | 1.00 |
| Primates | | *Pan troglodytes troglodytes* | 0.0079 | 2 | 0.42 | 0.29 | 0.03 | | 0.25 |
| Rodentia | | *Heliosciurus ruwenzorii* | 0.0077 | 2 | 2.06 | 0.43 | 0.01 | | 1.00 |
| Primates | | *Allochrocebus lhoesti* | 0.0068 | 2 | 1.18 | 0.29 | 0.01 | | 0.50 |
| Cetartiodactyla | | *Neotragus batesi batesi* | 0.0063 | 2 | 0.40 | 0.14 | 0.02 | | 1.00 |
| Primates | | *Colobus angolensis cottoni* | 0.0061 | 2 | 1.00 | 0.29 | 0.01 | | 1.00 |
| Primates | | *Cercopithecus hamlyni* | 0.0059 | 2 | 1.20 | 0.14 | 0.01 | | 0.50 |
| Primates | | *Cercocebus chrysogaster* | 0.0058 | 2 | 0.96 | 0.14 | 0.01 | | 1.00 |
| Cetartiodactyla | | *Cephalophus ogilbyi crusalbum* | 0.0058 | 1 | 1.15 | 0.29 | 0.01 | | 1.00 |
| Primates | | *Pan paniscus* | 0.0053 | 1 | 0.48 | 0.71 | 0.02 | | **0.25** |
| Primates | | *Euoticus pallidus talboti* | 0.0052 | 1 | 2.08 | 0.14 | 0.00 | | 1.00 |
| Lagomorpha | | *Poelagus marjorita* | 0.0052 | 1 | 1.38 | 0.29 | 0.01 | | 1.00 |
| Rodentia | | *Funisciurus carruthersi* | 0.0051 | 1 | 2.08 | 0.14 | 0.00 | | 1.00 |
| Primates | | *Cercopithecus mitis mitis* | 0.0051 | 1 | 1.85 | 0.14 | 0.00 | | - |
| Primates | | *Galago matschiei* | 0.0050 | 1 | 2.17 | 0.14 | 0.00 | | 1.00 |
| Primates | | *Sciurocheirus alleni cameronensis* | 0.0050 | 1 | 2.00 | 0.29 | 0.00 | | 1.00 |
| Carnivora | | *Genetta poensis* | 0.0045 | 1 | 1.32 | 0.14 | 0.01 | |  |
| Primates | | *Lophocebus opdenboschi* | 0.0041 | 1 | 1.09 | 0.14 | 0.01 | | **0.50** |
| Carnivora | | *Genetta cristata* | 0.0039 | 1 | 1.47 | 0.14 | 0.00 | | **0.50** |
| Primates | | *Cercopithecus pogonias pogonias* | 0.0037 | 1 | 1.30 | 0.14 | 0.00 | | 0.50 |
| Proboscidea | | *Loxodonta cyclotis* | 0.0034 | 1 | 0.18 | 0.43 | 0.03 | | 0.50 |
| Primates | | *Arctocebus calabarensis* | 0.0034 | 1 | 0.86 | 0.29 | 0.01 | | 1.00 |
| Afrosoricida | | *Micropotamogale ruwenzorii* | 0.0033 | 1 | 2.38 | 0.14 | 0.00 | | 0.75 |
| Primates | | *Procolobus rufomitratus lulindicus* | 0.0029 | 1 | 1.12 | 0.29 | 0.00 | | 1.00 |
| Primates | | *Cercopithecus erythrotis camerunensis* | 0.0029 | 1 | 2.03 | 0.14 | 0.00 | | **0.50** |
| Primates | | *Cercopithecus mitis heymansi* | 0.0025 | 1 | 1.85 | 0.14 | 0.00 | | 0.75 |
| Carnivora | | *Bdeogale jacksoni* | 0.0023 | 1 | 1.38 | 0.29 | 0.00 | | 0.75 |
| Cetartiodactyla | | *Neotragus batesi harrisoni* | 0.0022 | 1 | 0.40 | 0.14 | 0.01 | | 1.00 |
| Primates | | *Procolobus rufomitratus langi* | 0.0021 | 1 | 1.08 | 0.29 | 0.00 | | 1.00 |
| Primates | | *Cercopithecus mitis doggeti* | 0.0021 | 1 | 1.85 | 0.14 | 0.00 | | 1.00 |
| Carnivora | | *Genetta victoriae* | 0.0021 | 1 | 0.15 | 0.14 | 0.02 | | 1.00 |
| Cetartiodactyla | | *Cephalophus ogilbyi ogilbyi* | 0.0020 | 1 | 1.15 | 0.29 | 0.00 | | **0.50** |
| Cetartiodactyla | | *Okapia johnstoni* | 0.0020 | 1 | 0.31 | 0.14 | 0.01 | | 0.25 |
| Primates | | *Cercopithecus wolfi pyrogaster* | 0.0020 | 1 | 1.30 | 0.14 | 0.00 | | 1.00 |
| Primates | | *Pan troglodytes ellioti* | 0.0019 | 1 | 0.42 | 0.29 | 0.01 | | **0.25** |
| Primates | | *Colobus guereza matschiei* | 0.0018 | 1 | 2.00 | 0.29 | 0.00 | | - |
| Primates | | *Colobus angolensis cordeiri* | 0.0018 | 1 | 1.00 | 0.29 | 0.00 | | 0.75 |
| Primates | | *Cercopithecus ascanius atrinasus* | 0.0017 | 1 | 2.03 | 0.29 | 0.00 | | - |
| Primates | | *Cercopithecus wolfi elegans* | 0.0015 | 1 | 1.30 | 0.14 | 0.00 | | 0.75 |
| Cetartiodactyla | | *Tragelaphus derbianus* | 0.0013 | 1 | 0.13 | 0.29 | 0.01 | | 1.00 |
| Primates | | *Cercopithecus sclateri* | 0.0013 | 1 | 1.32 | 0.14 | 0.00 | | **0.50** |
| Primates | | *Lophocebus ugandae* | 0.0010 | 1 | 1.20 | 0.29 | 0.00 | | 1.00 |
| Primates | | *Mandrillus leucophaeus leucophaeus* | 0.0010 | 1 | 0.86 | 0.29 | 0.00 | | **0.25** |
| Primates | | *Procolobus rufomitratus foai* | 0.0008 | 1 | 1.05 | 0.29 | 0.00 | | 1.00 |
| Primates | | *Cercopithecus lomamiensis* | 0.0006 | 1 | 1.14 | 0.14 | 0.00 | | **0.50** |
| Rodentia | | *Paraxerus cooperi* | 0.0006 | 1 | 2.11 | 0.14 | 0.00 | | - |
| Primates | | *Procolobus rufomitratus parmentieri* | 0.0006 | 1 | 1.18 | 0.29 | 0.00 | | 1.00 |
| Primates | | *Procolobus rufomitratus ellioti* | 0.0006 | 1 | 1.01 | 0.29 | 0.00 | | 1.00 |
| Primates | | *Gorilla beringei graueri* | 0.0005 | 1 | 0.32 | 0.14 | 0.00 | | 0.25 |
| Primates | | *Allochrocebus preussi pre* | 0.0005 | 1 | 1.20 | 0.14 | 0.00 | | **0.25** |
| Primates | | *Allochrocebus solatus* | 0.0004 | 1 | 1.14 | 0.14 | 0.00 | | **0.50** |
| Primates | | *Colobus angolensis ruwenzorii* | 0.0003 | 1 | 1.00 | 0.29 | 0.00 | | 0.50 |
| Rodentia | | *Funisciurus duchaillui* | 0.0002 | 1 | 2.18 | 0.14 | 0.00 | | - |
| Primates | | *Procolobus rufomitratus tephrosceles* | 0.0001 | 1 | 1.21 | 0.29 | 0.00 | | **0.25** |
| Primates | | *Sciurocheirus alleni alleni* | 0.0001 | 1 | 2.00 | 0.29 | 0.00 | | **0.25** |
| Primates | | *Euoticus pallidus pallidus* | 0.0001 | 1 | 2.08 | 0.14 | 0.00 | | **0.25** |
| Primates | | *Lophocebus osmani* | 0.0001 | 1 | 1.20 | 0.29 | 0.00 | | 1.00 |
| Primates | | *Cercopithecus erythrotis erythrotis* | 0.0001 | 1 | 2.03 | 0.14 | 0.00 | | **0.50** |
| Primates | | *Colobus satanas satanas* | 0.0000 | 1 | 1.44 | 0.14 | 0.00 | | **0.25** |
| Primates | | *Colobus angolensis prigoginei* | 0.0000 | 1 | 1.00 | 0.29 | 0.00 | | **0.25** |
| Primates | | *Mandrillus leucophaeus poensis* | 0.0000 | 1 | 0.86 | 0.29 | 0.00 | | **0.25** |
| Primates | | *Allochrocebus preussi ins* | 0.0000 | 1 | 1.20 | 0.14 | 0.00 | | **0.25** |
| Primates | | *Procolobus pennantii pennantii* | 0.0000 | 1 | 1.00 | 0.14 | 0.00 | | **0.25** |
| Primates | | *Procolobus preussi* | 0.0000 | 1 | 1.06 | 0.14 | 0.00 | | **0.00** |
| Primates | | *Procolobus pennantii bouvieri* | 0.0000 | 1 | 0.95 | 0.14 | 0.00 | | **0.00** |
| Primates | | *Cercopithecus dryas* | 0.0000 | 1 | 1.35 | 0.14 | 0.00 | | **0.00** |
| Primates | | *Cercopithecus mitis schoutedeni* | 0.0000 | 1 | 1.85 | 0.14 | 0.00 | | **0.00** |
| Primates | | *Gorilla gorilla diehli* | 0.0000 | 1 | 0.20 | 0.14 | 0.00 | | **0.00** |
| Primates | | *Gorilla gorilla gorilla* | 0.0000 | 1 | 0.20 | 0.14 | 0.03 | | 0.00 |
| Primates | | *Gorilla beringei beringei* | 0.0000 | 1 | 0.32 | 0.14 | 0.00 | | **0.00** |
| Perissodactyla | | *Diceros bicornis* | 0.0000 | 1 | 0.05 | 0.57 | 0.20 | | **0.00** |

**Appendix S2. Predictor variables (and primary sources) used to construct favorability models for all species in our study**: A species’ distribution is influenced by multiple causal factors such as climate, topography, human activity, history, and the population dynamics of the species itself, among others (Lomolino *et al*., 2005; Guisan & Thuiller, 2005; Márquez *et al*., 2011). Climate, topo-hydrography and land cover/land use, in particular, have a dominant influence on species distributions at scales ranging from site (10-1000 m) to global levels (>10000 km) (Mackey & Lindenmayer, 2001; Pearson & Dawson, 2005). However, the inclusion of anthropogenic variables in distribution modeling is also important, since human presence is a primary factor impacting species distributions (Corsi *et al*., 1999). Spatial descriptors need also to be included in the variable set to account for autocorrelation, and because it may explain the effect of dispersal barriers, geological history and biotic interactions (Legendre, 1993). For further details on variable building and data sources see Appendix S3.

Climate (WorldClim, IPCC5/CMIP5) (representative of 1950-2000)

- Maximum temperature of July
- Minimum temperature of January
- Annual temperature range
- Annual precipitation
- Pluviometric irregularity

Topography (GlobDEM50; GTOPO30)

- Elevation
- Slope

Hydrography (HydroSHEDS; Global Land Cover 2000)

- Distance to water masses
- Distance to minor rivers

Land cover / land use

- Forest (Collection 5 MODIS Global Land Cover Type, which processed data for each year for the period 2001-2005)
- Grassland-shrubland-savanna (Collection 5 MODIS Global Land Cover Type)
- Woody savanna (Collection 5 MODIS Global Land Cover Type)
- Cropland-natural vegetation mosaic (Collection 5 MODIS Global Land Cover Type)
- Desert (Collection 5 MODIS Global Land Cover Type)
- Occurrence of cropland (FGGD Digital Atlas for the year 2000)
- Occurrence of pasture and browse (FGGD Digital Atlas for the year 2000)
- Intact forest (IFL) in 2000

Other anthropogenic pressure variables

- Percentage of area equipped for irrigation (Global Map of Irrigation Areas v.4.0.1) around the year 2000
- Global climate, soil and terrain slope constraints for cropping activities (FAO & IIASA, 2007)
- Density of poultry farms (FAO's Gridded Livestock of the World, derived from various national census reports, livestock surveys and data archives from 1992 to 2003)
- Density of pigs (FAO's Gridded Livestock of the World)
- Density of cattle (FAO's Gridded Livestock of the World)
- Density of small ruminants (FAO's Gridded Livestock of the World)
- Distance to roads (FAO/GIS, DCW) in 2002
- Distance to rail-roads (FAO/GIS, DCW) in 2002
- Distance to populated places (RWDB2) in 2000
- Rural population density (LandScan™ 2008 High Resolution Global Population Data Set; MODIS 500-m Map of Global Urban Extent, produced using data circa 2001-2002)

Spatial descriptors: these two variables were combined, following the trend surface approach described in the main text.

- Geographic latitude
- Geographic longitude

**References**

Corsi, F., Duprè, E., Boitani, L. (1999) A large-scale model of wolf distribution in Italy for conservation planning. *Conservation Biology* **13**:150-159.

FAO and IIASA (2007). *Mapping Biophysical Factors that Influence Agricultural Production and Rural Vulnerability* [van Velthuizen, H., Huddleston, B., Fischer, G., Salvatore, M., Ataman, E., Nachtergaele, F., Zanetti, M., Bloise, M.]. Environmental and Natural Resources Series No. 11. Rome.

Guisan, A., Thuiller, W. (2005) Predicting species distribution: offering more than simple habitat models. *Ecology Letters* **8**: 993-1009.

Legendre, P. (1993) Spatial autocorrelation: trouble or new paradigm? *Ecology* **74**:1659-1673.

Lomolino, M.V., Riddle, B.R., Brown, J.H. (2005) Distributions of species. In: Lomolino, M.V., Riddle, B.R., Brown, J.H. (eds) *Biogeography*, 3rd edn. Sinauer, Sunderland, pp 65-96.

Mackey, B.G., Lindenmayer, D.B. (2001) Towards a hierarchical framework for modelling the spatial distribution of animals. *Journal of Biogeography* **28**: 1147-1166.

Márquez, A.L., Real, R., Olivero, J., Estrada, A. (2011) Combining climate with other influential factors for modelling the impact of climate change on species distribution. *Climatic Change* **108**: 135-157.

Pearson, R.G., Dawson, T.P. (2005) Predicting the impacts of climate change on the distribution of species: are bioclimate envelope models useful? *Global Ecology & Biogeography* **12**: 361-371.

**Appendix S3. Detailed description of predictor variable design**.

*Climate*

Five climate variables were used: "maximum temperature of July", "minimum temperature of January", "annual temperature range", "annual precipitation", and "pluviometric irregularity" ―i.e. annual variation coefficient of monthly precipitation. We built these variables using 30 arc-second**-**resolution layers for maximum temperature of July, minimum temperature of January and monthly precipitation regarding current conditions (~1950-2000), which were downloaded from WorldClim (http://www.worldclim.org). These measures were chosen as variable sources in order to match with the set of variables with available predictions for 2050.

*Topography*

"Elevation" and "slope" values for model training, including the entire African continent, were derived from GTOPO30 (US Geological Survey, 1996), with a 30 arc-second**-**resolution. The GlobDEM50 3-arc-second-resolution digital elevation model, based on raw data from the Shuttle Radar Topography Mission (SRTM) (Farr & Kobrick, 2000), was alternatively used for model downscaling within our study area.

*Hydrography*

Two variables measuring "distance to water masses" and "distance to minor rivers" were calculated using the hydrologically conditioned 3-arc-second-resolution elevation layer distributed with HydroSHEDS ([http://hydrosheds.cr.usgs.gov](http://hydrosheds.cr.usgs.gov/)). Water masses were referred to as lakes (delimited as in http://www.naturalearthdata.com) and main river courses, which were defined as river courses whose water flow was higher than 10^6^ according to HydroSHEDS. These rivers included Zambezi, Limpopo, Cubango, Nile, Niger, Congo, Senegal, Chari, Orange, and some principal tributaries. Instead, we considered minor rivers to be those ones whose water flow was between 10^4^ and 10^6^, which included most tributaries and other main courses not cited above. Rivers outlined within the Sahara Desert in HydroSHEDS were eliminated from the data set by intersecting with the Bare Areas (19) land cover class in Global Land Cover 2000 (Bartholomé & Belward, 2005). This layer was chosen because it allowed respecting river courses running across the desert, as Nile and Niger.

*Land cover / land use*

Land-cover / land use variable describing the proportion of surface area covered with the corresponding classes within each cell were calculated (see Romero *et al*., 2012).

Five variables were extracted from the Collection 5 MODIS Global Land Cover Type product, which processed data for each year during the period 2001-2005 (Friedl *et al*., 2010): "forest" ―land-cover classes 1 to 5―, "grassland-shrubland-savanna" ―classes 6, 7, 9 and 10―, "woody savanna" ―class 8―, "cropland-natural vegetation mosaic" ―class14― and "desert" ―class 16.

The source for the "occurrence of cropland" and for the "occurrence of pasture and browse" was the Food Insecurity, Poverty and Environment Global GIS Database (FGGD Digital Atlas for the year 2000) (FAO & IIASA, 2007).

"Intact forest" surface was calculated using the World Intact Forest Landscapes (IFL) Map in the year 2000 (Potapov *et al*., 2008).

*Other anthropogenic pressure variables*

Two more agriculture variables were included in the analysis. The "percentage of area equipped for irrigation" was got from the Global Map of Irrigation Areas (version 4.0.1) around the year 2000 (http://www.fao.org/nr/water); maps to calculate the area with "global climate, soil and terrain slope constraints for cropping activities" was extracted from FAO & IIASA (2007).

Livestock was represented by four variables: "density of poultry farms", "density of pigs", "density of cattle" and "density of small ruminants". Data were extracted from FAO's Gridded Livestock of the World maps, derived from various national census reports, livestock surveys and data archives from 1992 to 2003 (Robinson *et al*., 2007).

We calculated "distance to roads" and "distance to rail-roads" with ArcGIS 10.0, using the maps extracted by FAO/GIS (http://www.fao.org/geonetwork) from Vector Map Level 0 at the Digital Chart of the World (DCW, [http://worldmap.harvard.edu](http://worldmap.harvard.edu/)), updated in 2002.

The "distance to populated places" was calculated with ArcGIS 10.0, using the Administrative Centres & Populated Places shapefile at the Relational World Database II (RWDB2) updated in 2000 (http://www.fao.org/geonetwork) as a reference.

"Rural population density" was estimated by combining two data sources: (1) population density, calculated utilizing the LandScan™ 2008 High Resolution Global Population Data Set (copyrighted by UT-Battelle, LLC, operator of Oak Ridge National Laboratory); and (2) urban areas, taken from MODIS 500-m Map of Global Urban Extent (produced using data circa 2001-2002, see Schneider *et al*., 2009, 2010). Using ArcGIS 10.0, population density was valued as 0 within a 2-km buffer around urban areas.

*How variables were prepared for modeling*

Values of all the above-described variables had, initially, spatial resolutions finer than 0.1º x 0.1º. We then calculated variable values for all 1º x 1º-resolution grid cells in Africa, and for all 0.1º x 0.1º-resolution grid cells in our study area (see main text). The former were used for favorability-model training, whereas the latter were used for model downscaling. These variable values were estimated as the average of the initial values within each cell, for which we used Zonal Statistic tools of ArcGIS 10.0.

**References**

Bartholomé, E., Belward, A. S. (2005). GLC2000: a new approach to global land cover mapping from Earth observation data. *International Journal of Remote Sensing* **26**:1959-1977.

FAO and IIASA (2007). *Mapping Biophysical Factors that Influence Agricultural Production and Rural Vulnerability* [van Velthuizen, H., Huddleston, B., Fischer, G., Salvatore, M., Ataman, E., Nachtergaele, F., Zanetti, M., Bloise, M.]. Environmental and Natural Resources Series No. 11. Rome.

Farr, T. G., Kobrick, M. (2000). Shuttle Radar Topography Mission produces a wealth of data. *Transactions of the American Geophysical Union* **81**:583-585.

Friedl, M. A., Sulla-Menashe, D., Tan, B., Schneider, A., Ramankutty, N., Sibley, A., Huang, X. (2010). MODIS Collection 5 global land cover: Algorithm refinements and characterization of new datasets. *Remote Sensing of Environment* **114**:168-182.

Potapov, P., Yaroshenko, A., Turubanova, S., Dubinin, M., Laestadius, L., Thies, C., Aksenov, D., Egorov, A., Yesipova, Y., Glushkov, I., Karpachevskiy, M., Kostikova, A., Manisha, A., Tsybikova, E., Zhuravleva, I. (2008). Mapping the World's Intact Forest Landscapes by Remote Sensing. *Ecology and Society* **13**:51-66.

Robinson, T.P., Franceschini, G., Wint, W. (2007). The Food and Agriculture Organization's Gridded Livestock of the World. *Veterinaria Italiana* **43**:745-751.

Romero, D., Olivero, J., Real, R. (2012). Comparative assessment of different methods for using land-cover variables for distribution modelling of *Salamandra salamandra longirostris*. *Environmental Conservation* **40**:48-59.

Schneider, A., Friedl, M. A., Potere, D. (2009). A new map of global urban extent from MODIS data. *Environmental Research Letters* **4**:044003.

Schneider, A., Friedl, M. A., Potere, D. (2010). Monitoring urban areas globally using MODIS 500m data: New methods and datasets based on urban ecoregions. *Remote Sensing of Environment* **114**:1733-1746.

US Geological Survey (1996). *GTOPO30*. Land processes distributed active archive center (LP DAAC), EROS data center. https://lta.cr.usgs.gov/GTOPO30

**Appendix S4**. Number (N) and percentage (%) of taxa (species and subspecies) included in the five sustainablility categories defined by potential hunting sustainability (*PHS*). Sustainability increases from 1 to 5.

| **Permissive weighting** |  |  |  |  |  |  |  |
| --- | --- | --- | --- | --- | --- | --- | --- |
| **Family** |  | **1** | **2** | **3** | **4** | **5** | **Total** |
| Procaviidae | N | 0 | 0 | 0 | 2 | 1 | 3 |
|  | % | 0.0 | 0.0 | 0.0 | 66.7 | 33.3 | 100.0 |
| Elephantidae | N | 1 | 1 | 0 | 0 | 0 | 2 |
|  | % | 50.0 | 50.0 | 0.0 | 0.0 | 0.0 | 100.0 |
| Tenrecidae | N | 0 | 0 | 0 | 2 | 0 | 2 |
|  | % | 0.0 | 0.0 | 0.0 | 100.0 | 0.0 | 100.0 |
| Orycteropodidae | N | 0 | 1 | 0 | 0 | 0 | 1 |
|  | % | 0.0 | 100.0 | 0.0 | 0.0 | 0.0 | 100.0 |
| Hominidae | N | 8 | 0 | 0 | 0 | 0 | 8 |
|  | % | 100.0 | 0.0 | 0.0 | 0.0 | 0.0 | 100.0 |
| Cercopithecidae | N | 13 | 19 | 9 | 24 | 7 | 72 |
|  | % | 18.1 | 26.4 | 12.5 | 33.3 | 9.7 | 100.0 |
| Lorisidae | N | 0 | 4 | 0 | 0 | 1 | 5 |
|  | % | 0.0 | 80.0 | 0.0 | 0.0 | 20.0 | 100.0 |
| Galagidae | N | 0 | 2 | 0 | 1 | 9 | 12 |
|  | % | 0.0 | 16.7 | 0.0 | 8.3 | 75.0 | 100.0 |
| Sciuridae | N | 2 | 1 | 0 | 0 | 18 | 21 |
|  | % | 9.5 | 4.8 | 0.0 | 0.0 | 85.7 | 100.0 |
| Nesomyidae | N | 0 | 0 | 0 | 1 | 1 | 2 |
|  | % | 0.0 | 0.0 | 0.0 | 50.0 | 50.0 | 100.0 |
| Anomaluridae | N | 1 | 0 | 0 | 1 | 1 | 3 |
|  | % | 33.3 | 0.0 | 0.0 | 33.3 | 33.3 | 100.0 |
| Hystricidae | N | 0 | 2 | 0 | 1 | 0 | 3 |
|  | % | 0.0 | 66.7 | 0.0 | 33.3 | 0.0 | 100.0 |
| Thryonomyidae | N | 0 | 0 | 0 | 1 | 0 | 1 |
|  | % | 0.0 | 0.0 | 0.0 | 100.0 | 0.0 | 100.0 |
| Leporidae | N | 0 | 0 | 0 | 1 | 0 | 1 |
|  | % | 0.0 | 0.0 | 0.0 | 100.0 | 0.0 | 100.0 |
| Canidae | N | 1 | 0 | 0 | 0 | 0 | 1 |
|  | % | 100.0 | 0.0 | 0.0 | 0.0 | 0.0 | 100.0 |
| Mustelidae | N | 1 | 1 | 1 | 2 | 1 | 6 |
|  | % | 16.7 | 16.7 | 16.7 | 33.3 | 16.7 | 100.0 |
| Nandiniidae | N | 0 | 1 | 0 | 0 | 0 | 1 |
|  | % | 0.0 | 100.0 | 0.0 | 0.0 | 0.0 | 100.0 |
| Felidae | N | 3 | 2 | 0 | 0 | 0 | 5 |
|  | % | 60.0 | 40.0 | 0.0 | 0.0 | 0.0 | 100.0 |
| Viverridae | N | 5 | 3 | 0 | 2 | 0 | 10 |
|  | % | 50.0 | 30.0 | 0.0 | 20.0 | 0.0 | 100.0 |
| Hyaenidae | N | 1 | 0 | 0 | 0 | 0 | 1 |
|  | % | 100.0 | 0.0 | 0.0 | 0.0 | 0.0 | 100.0 |
| Herpestidae | N | 2 | 3 | 1 | 6 | 2 | 14 |
|  | % | 14.3 | 21.4 | 7.1 | 42.9 | 14.3 | 100.0 |
| Manidae | N | 0 | 1 | 1 | 1 | 0 | 3 |
|  | % | 0.0 | 33.3 | 33.3 | 33.3 | 0.0 | 100.0 |
| Equidae | N | 1 | 0 | 0 | 0 | 0 | 1 |
|  | % | 100.0 | 0.0 | 0.0 | 0.0 | 0.0 | 100.0 |
| Rhinocerotidae | N | 1 | 0 | 0 | 0 | 0 | 1 |
|  | % | 100.0 | 0.0 | 0.0 | 0.0 | 0.0 | 0.0 |
| Suidae | N | 3 | 1 | 0 | 0 | 0 | 4 |
|  | % | 75.0 | 25.0 | 0.0 | 0.0 | 0.0 | 100.0 |
| Hippopotamidae | N | 1 | 0 | 0 | 0 | 0 | 1 |
|  | % | 100.0 | 0.0 | 0.0 | 0.0 | 0.0 | 100.0 |
| Tragulidae | N | 0 | 1 | 0 | 0 | 0 | 1 |
|  | % | 0.0 | 100.0 | 0.0 | 0.0 | 0.0 | 100.0 |
| Giraffidae | N | 1 | 0 | 0 | 0 | 0 | 1 |
|  | % | 100.0 | 0.0 | 0.0 | 0.0 | 0.0 | 100.0 |
| Bovidae | N | 7 | 12 | 0 | 3 | 0 | 22 |
|  | % | 31.8 | 54.5 | 0.0 | 13.6 | 0.0 | 100.0 |
| **Restrictive weighting** |  |  |  |  |  |  |  |
| **Family** |  | **1** | **2** | **3** | **4** | **5** | **Total** |
| Procaviidae | N | 0 | 0 | 1 | 1 | 1 | 3 |
|  | % | 0.0 | 0.0 | 33.3 | 33.3 | 33.3 | 100.0 |
| Elephantidae | N | 1 | 1 | 0 | 0 | 0 | 2 |
|  | % | 50.0 | 50.0 | 0.0 | 0.0 | 0.0 | 100.0 |
| Tenrecidae | N | 1 | 0 | 0 | 0 | 1 | 2 |
|  | % | 50.0 | 0.0 | 0.0 | 0.0 | 50.0 | 100.0 |
| Orycteropodidae | N | 0 | 0 | 0 | 0 | 1 | 1 |
|  | % | 0.0 | 0.0 | 0.0 | 0.0 | 100.0 | 100.0 |
| Hominidae | N | 6 | 2 | 0 | 0 | 0 | 8 |
|  | % | 75.0 | 25.0 | 0.0 | 0.0 | 0.0 | 100.0 |
| Cercopithecidae | N | 35 | 30 | 1 | 5 | 1 | 72 |
|  | % | 48.6 | 41.7 | 1.4 | 6.9 | 1.4 | 100.0 |
| Lorisidae | N | 1 | 4 | 0 | 0 | 0 | 5 |
|  | % | 20.0 | 80.0 | 0.0 | 0.0 | 0.0 | 100.0 |
| Galagidae | N | 5 | 2 | 0 | 0 | 5 | 12 |
|  | % | 41.7 | 16.7 | 0.0 | 0.0 | 41.7 | 100.0 |
| Sciuridae | N | 3 | 9 | 0 | 7 | 2 | 21 |
|  | % | 14.3 | 42.9 | 0.0 | 33.3 | 9.5 | 100.0 |
| Nesomyidae | N | 0 | 0 | 0 | 0 | 2 | 2 |
|  | % | 0.0 | 0.0 | 0.0 | 0.0 | 100.0 | 100.0 |
| Anomaluridae | N | 0 | 1 | 0 | 1 | 1 | 3 |
|  | % | 0.0 | 33.3 | 0.0 | 33.3 | 33.3 | 100.0 |
| Hystricidae | N | 0 | 0 | 0 | 2 | 1 | 3 |
|  | % | 0.0 | 0.0 | 0.0 | 66.7 | 33.3 | 100.0 |
| Thryonomyidae | N | 0 | 0 | 0 | 0 | 1 | 1 |
|  | % | 0.0 | 0.0 | 0.0 | 0.0 | 100.0 | 100.0 |
| Leporidae | N | 1 | 0 | 0 | 0 | 0 | 1 |
|  | % | 100.0 | 0.0 | 0.0 | 0.0 | 0.0 | 100.0 |
| Canidae | N | 0 | 1 | 0 | 0 | 0 | 1 |
|  | % | 0.0 | 100.0 | 0.0 | 0.0 | 0.0 | 100.0 |
| Mustelidae | N | 0 | 2 | 0 | 0 | 4 | 6 |
|  | % | 0.0 | 33.3 | 0.0 | 0.0 | 66.7 | 100.0 |
| Nandiniidae | N | 0 | 0 | 0 | 1 | 0 | 1 |
|  | % | 0.0 | 0.0 | 0.0 | 100.0 | 0.0 | 100.0 |
| Felidae | N | 0 | 3 | 0 | 0 | 2 | 5 |
|  | % | 0.0 | 60.0 | 0.0 | 0.0 | 40.0 | 100.0 |
| Viverridae | N | 3 | 3 | 0 | 1 | 3 | 10 |
|  | % | 30.0 | 30.0 | 0.0 | 10.0 | 30.0 | 100.0 |
| Hyaenidae | N | 0 | 1 | 0 | 0 | 0 | 1 |
|  | % | 0.0 | 100.0 | 0.0 | 0.0 | 0.0 | 100.0 |
| Herpestidae | N | 1 | 7 | 0 | 1 | 5 | 14 |
|  | % | 7.1 | 50.0 | 0.0 | 7.1 | 35.7 | 100.0 |
| Manidae | N | 0 | 1 | 0 | 1 | 1 | 3 |
|  | % | 0.0 | 33.3 | 0.0 | 33.3 | 33.3 | 100.0 |
| Equidae | N | 0 | 1 | 0 | 0 | 0 | 1 |
|  | % | 0.0 | 100.0 | 0.0 | 0.0 | 0.0 | 100.0 |
| Rhinocerotidae | N | 1 | 0 | 0 | 0 | 0 | 1 |
|  | % | 100.0 | 0.0 | 0.0 | 0.0 | 0.0 | 0.0 |
| Suidae | N | 0 | 3 | 0 | 1 | 0 | 4 |
|  | % | 0.0 | 75.0 | 0.0 | 25.0 | 0.0 | 100.0 |
| Hippopotamidae | N | 0 | 1 | 0 | 0 | 0 | 1 |
|  | % | 0.0 | 100.0 | 0.0 | 0.0 | 0.0 | 100.0 |
| Tragulidae | N | 0 | 1 | 0 | 0 | 0 | 1 |
|  | % | 0.0 | 100.0 | 0.0 | 0.0 | 0.0 | 100.0 |
| Giraffidae | N | 1 | 0 | 0 | 0 | 0 | 1 |
|  | % | 100.0 | 0.0 | 0.0 | 0.0 | 0.0 | 100.0 |
| Bovidae | N | 4 | 15 | 1 | 1 | 1 | 22 |
|  | % | 18.2 | 68.2 | 4.5 | 4.5 | 4.5 | 100.0 |
